# The E3 ubiquitin ligase ITCH negatively regulates intercellular communication via gap junctions by targeting connexin43 for lysosomal degradation

**DOI:** 10.1007/s00018-024-05165-8

**Published:** 2024-04-10

**Authors:** Max Zachrisson Totland, Lars Mørland Knudsen, Nikoline Lander Rasmussen, Yasufumi Omori, Vigdis Sørensen, Vilde C. Wivestad Elster, Jakob Mørkved Stenersen, Mathias Larsen, Caroline Lunder Jensen, Anna A. Zickfeldt Lade, Emilie Bruusgaard, Sebastian Basing, Kushtrim Kryeziu, Andreas Brech, Trond Aasen, Ragnhild A. Lothe, Edward Leithe

**Affiliations:** 1 Department of Molecular Oncology, Institute for Cancer Research, Oslo University Hospital, Oslo, NO-0424 Norway; 2 Department of Core Facilities, Institute for Cancer Research, Oslo University Hospital, Oslo, NO-0424 Norway; 3 Department of Molecular Cell Biology, Institute for Cancer Research, Oslo University Hospital, Oslo, 0379 Norway; 4 Department of Biosciences, Faculty of Mathematics and Natural Sciences, Oslo, 0316 Norway; 5 Centre for Cancer Cell Reprogramming, Faculty of Medicine, University of Oslo, Oslo, 0379 Norway; 6 Institute of Clinical Medicine, Faculty of Medicine, University of Oslo, Oslo, 0317 Norway; 7 Department of Molecular and Tumour Pathology, Akita University Graduate School of Medicine, Akita, 010-8543 Japan; 8 Patologia Molecular Translacional, Vall d’Hebron Institut de Recerca (VHIR), Vall d’Hebron Hospital Universitari, Vall d’Hebron Barcelona Hospital Campus, Passeig Vall d’Hebron 119-129, Barcelona, 08035 Spain; 9 Present Address: Centre for Molecular Medicine Norway, Faculty of Medicine, Oslo, Norway

**Keywords:** Cervix, Connexin, Deubiquitination, Endosome, NEDD4

## Abstract

Intercellular communication via gap junctions has a fundamental role in regulating cell growth and tissue homeostasis, and its dysregulation may be involved in cancer development and radio- and chemotherapy resistance. Connexin43 (Cx43) is the most ubiquitously expressed gap junction channel protein in human tissues. Emerging evidence indicates that dysregulation of the sorting of Cx43 to lysosomes is important in mediating the loss of Cx43-based gap junctions in cancer cells. However, the molecular basis underlying this process is currently poorly understood. Here, we identified the E3 ubiquitin ligase ITCH as a novel regulator of intercellular communication via gap junctions. We demonstrate that ITCH promotes loss of gap junctions in cervical cancer cells, which is associated with increased degradation of Cx43 in lysosomes. The data further indicate that ITCH interacts with and regulates Cx43 ubiquitination and that the ITCH-induced loss of Cx43-based gap junctions requires its catalytic HECT (homologous to E6-AP C-terminus) domain. The data also suggest that the ability of ITCH to efficiently promote loss of Cx43-based gap junctions and degradation of Cx43 depends on a functional PY (PPXY) motif in the C-terminal tail of Cx43. Together, these data provide new insights into the molecular basis underlying the degradation of Cx43 and have implications for the understanding of how intercellular communication via gap junctions is lost during cancer development.

**Supplementary Information:**

The online version contains supplementary material available at 10.1007/s00018-024-05165-8.

## Introduction

Gap junctions consist of clusters of intercellular channels in the plasma membrane that enable direct cell-to-cell movement of ionic currents and signaling molecules ( < ~ 1.2 kDa) [1, 2]. A core function of gap junctions is to assist groups of cells to meet metabolic demands and maintain cellular homeostasis. The fundamental units of the gap junctions are integral membrane proteins termed connexins [1, 2]. The human genome encodes 21 connexin proteins, among which the most commonly expressed member is connexin43 (Cx43). The connexins assemble into hexameric cylindrical structures known as connexons along their transport via the exocytic pathway to the plasma membrane. After arriving at the plasma membrane, connexons are able to dock head-to-head with connexons in neighboring cells and thereby form gap junction intercellular channels [1, 2]. Dysregulation of gap junction intercellular communication is associated with numerous diseases, including cancer [3–5]. Cx43 has tumor suppressor functions in multiple tissue types, and Cx43-based gap junctions are often lost during cancer development [3–5]. The absence of gap junctions formed by Cx43 may contribute to increased cancer cell growth and may also affect their response to radio- and chemotherapy. The loss of Cx43-based gap junctions in cancer cells can be due to genetic or epigenetic changes of the gene encoding Cx43 (*GJA1*) or to dysregulation of Cx43 post-translationally [3–5].

The connexin protein pool that constitutes the gap junctions of a cell is constantly renewed, as newly formed intercellular channels are continually added to the outer edges of gap junctions, whereas older channels are cleared from the center of the gap junction plaque through endocytosis [6–11]. In line with these findings, increasing experimental evidence suggests that intercellular communication via gap junctions can be regulated through modulation of the endocytosis and degradation rates of connexins [12–19]. The endocytosis of a gap junction results in the formation of a connexin-enriched, double-membrane vacuole called an annular gap junction, also known as a connexosome [20, 21]. Subsequently, Cx43 can be sorted to lysosomes via the endolysosomal or autophagosomal pathways or by fusion between annular gap junctions and lysosomes [22, 23]. Previous studies have suggested that aberrant endocytosis of Cx43 or deregulation of its trafficking along the endolysosomal pathway may be involved in mediating the loss of Cx43-based gap junctions in cancer cells [12, 24, 25]. Moreover, a variety of known or potential chemical carcinogens have been shown to induce endocytosis of Cx43-based gap junctions or to cause derailed post-endocytic sorting of Cx43 [26–30].

Post-translational modifications of connexins have key roles in the regulation of gap junction intercellular communication [31]. Although connexin phosphorylation has been extensively studied, the role of other post-translational modifications in the regulation of gap junctions is considerably less well understood [32, 33]. Ubiquitination of Cx43 has been suggested to have important roles in the regulation of gap junction endocytosis [15, 34–36], trafficking of Cx43 from early endosomes to lysosomes [37, 38], and autophagy-mediated degradation of Cx43 [34, 35]. Increasing evidence also suggests that endocytosis and degradation of Cx43-based gap junctions are controlled by complex crosstalk between Cx43 phosphorylation and ubiquitination [39–41]. Notwithstanding the growing body of experimental data suggesting that Cx43 ubiquitination is important in controlling gap junction intercellular communication, the understanding of the molecular basis underlying the catalysis of Cx43 ubiquitination remain fragmentary [42]. It has also been suggested that the regulation of gap junction levels does not involve Cx43 ubiquitination [43].

E3 ubiquitin ligases are the major regulatory determinants in protein ubiquitination [44]. The first E3 ubiquitin ligase demonstrated to bind to Cx43 and regulate its degradation was NEDD4 (neural precursor cell expressed developmentally down-regulated protein 4) [35, 45–47]. NEDD4 is the prototype member of the NEDD4 family of E3 ubiquitin ligases, which consists of nine members [48]. Subsequent studies indicated that two other NEDD4 family members, SMAD ubiquitination regulatory factor 2 (SMURF2) and WW domain-containing protein 1 (WWP1), are also involved in controlling Cx43 degradation [49–51]. Moreover, it was recently demonstrated that a fourth member of the NEDD4 family, NEDD4-2, regulates the ubiquitination and degradation of Cx43 in astrocytes [52]. In addition, the E3 ubiquitin ligase TRIM21 (cytosolic Fc receptor tripartite motif 21), a member of the tripartite motif family (TRIM) protein family, has been shown to interact with Cx43 and control its ubiquitination and degradation [53].

The E3 ubiquitin ligase ITCH is a member of the NEDD4 family. *Itch* knockout mice display a phenotype of debilitating immunological defects that ultimately lead to death at around six months of age [54]. ITCH deficiency has also been identified in humans, where a homozygous truncation of the *ITCH* gene results in syndromic multisystem autoimmune disease and developmental abnormalities in a group of Amish children [55]. While several of the known substrates of ITCH have important roles in immunological processes, the activity of ITCH is not limited to the immune system, and it is widely expressed across tissue types [56]. ITCH is involved in the regulation of several signaling pathways with important roles in tumorigenesis, including the Wnt/β-catenin, Hedgehog, Hippo, and Notch pathways [56]. It has been suggested to protect against cancer by negatively regulating the levels of proto-oncogenic proteins such as c-Jun, Cbl-b, and c-FLIP [57–59]. However, ITCH may also exert pro-tumorigenic functions through directly destabilizing tumor suppressors such as p63, p73, and LATS1 [60–62]. ITCH inhibition has been shown to augment the effect of chemotherapeutic drugs, suggesting that it is a potential pharmacological target in cancer therapy [63].

It has previously been shown that upregulation of ITCH is associated with the malignant transformation of the cervical epithelium [64]. In the present study, evidence is provided that ITCH interacts with Cx43 and regulates its ubiquitination in cervical cancer cells. The data indicate that ITCH negatively controls the size of gap junctions by targeting Cx43 for lysosomal degradation in a process that is dependent on its catalytic homologous to E6-AP carboxyl terminus (HECT) domain. The results also suggest that the interaction between ITCH and Cx43 involves a PY motif in the C-terminal tail of Cx43 and that the ability of ITCH to efficiently induce loss of Cx43-based gap junctions and degradation of Cx43 requires an intact PY motif. Taken together, the data identify ITCH as a novel regulator of gap junction intercellular communication and may have implications for our understanding of the post-translational mechanisms involved in mediating the loss of this form of cell-cell communication during cervical cancer pathogenesis.

## Results

### ITCH negatively regulates the level of Cx43-based gap junctions in cervical cancer cells

**Fig. 1:**
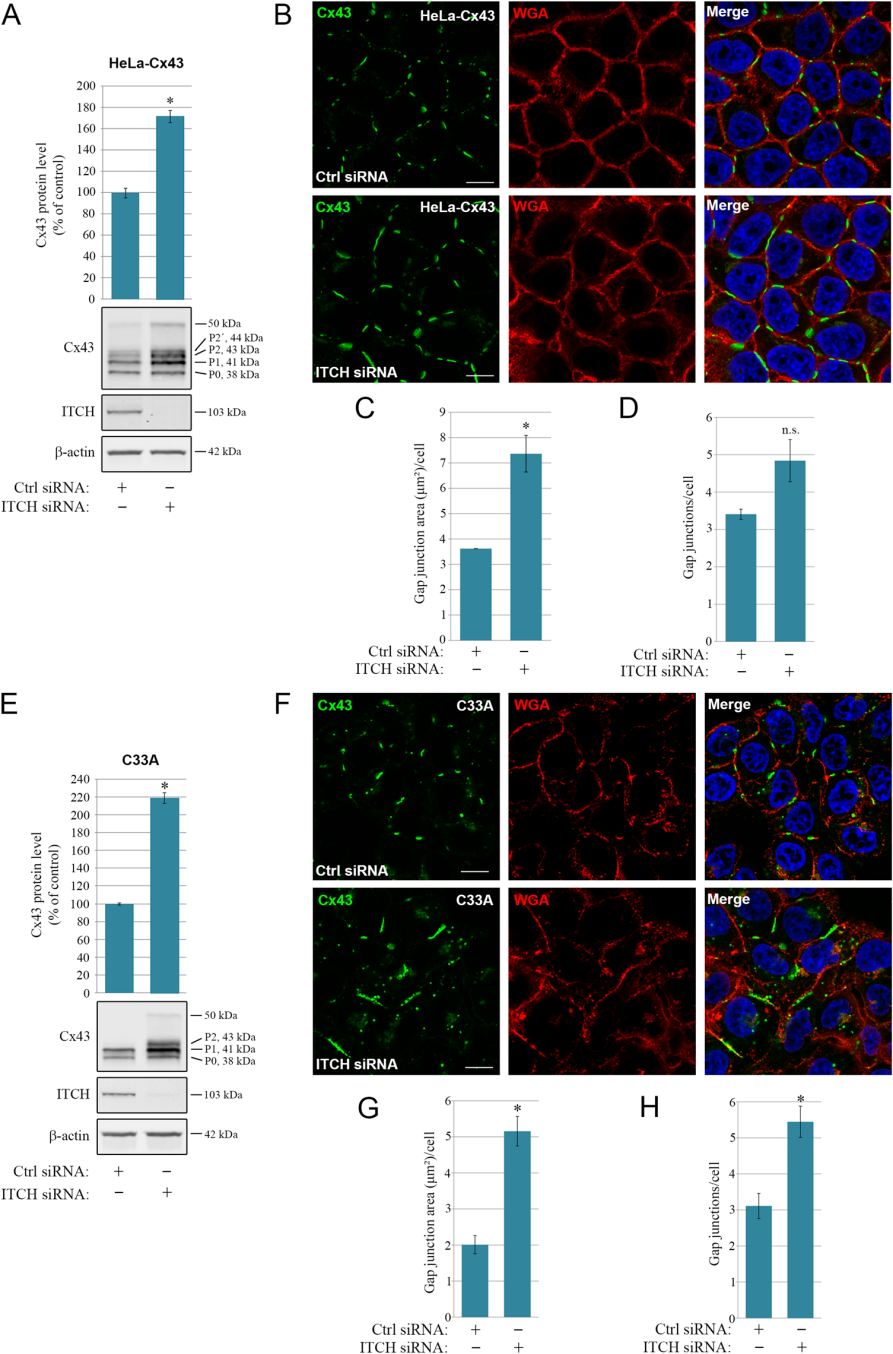
Role of ITCH in regulating the Cx43 protein level and gap junction size in HeLa-Cx43 and C33A cells. HeLa-Cx43 **(A)** or C33A **(E)** cells were transfected with control siRNA or with an siRNA sequence against ITCH (corresponding to ITCH #1 in Suppl. Figs. S1 and S2). Cell lysates were prepared 96 h after transfection, and equal amounts of total cell protein lysates were subjected to SDS-PAGE. ITCH, Cx43, and β-actin were detected by western blotting. The intensities of the Cx43 bands on western blots were quantified and normalized to the β-actin level. Values shown are the mean ± S.E.M. of four independent experiments. *p < 0.0005. Approximate molecular mass in kDa is indicated. HeLa-Cx43 **(B)** or C33A **(F)** cells were transfected with control siRNA or with an siRNA sequence against ITCH (corresponding to ITCH #1 in Suppl. Figs. S1 and S2) for 96 h. The cells were fixed and stained with anti-Cx43 (green) antibodies followed by Alexa488-conjugated secondary antibodies. The plasma membrane was stained with Alexa555-conjugated WGA (red). Nuclei were stained with Hoechst 33342 (blue). The cells were visualized by confocal fluorescence microscopy. Scale bar, 10 μm, applies to all images. **(C, D, G, H)** Quantification of the area of Cx43-based gap junctions per cell (C,G) and number of Cx43-based gap junctions per cell (D,H) based on confocal fluorescence microscopy images in B and F. Values shown are the means ± S.E.M. of three independent experiments. *p < 0.05. n.s., not significant

Cervical cancer pathogenesis is associated with the loss of Cx43-based gap junctions [65, 66]. We therefore consider HeLa and other cervical cancer cells as suitable model systems for studying the molecular mechanisms involved in the loss of intercellular communication via gap junctions in carcinogenesis. In search of potential E3 ubiquitin ligases that regulate Cx43 ubiquitination and degradation in HeLa cells stably transfected with Cx43 (HeLa-Cx43), we found that conducting a 96-hour transfection with an siRNA sequence targeting ITCH was associated with a strong increase in Cx43 protein levels compared with those after transfection with control siRNA (Fig. 1A). When detected by SDS-PAGE and western blotting, Cx43 was found to form several distinct bands, consistent with the results of previous studies (Fig. 1A) [33]. The three major Cx43 bands are commonly referred to as Cx43-P0, -P1, and -P2 [33]. Cx43-P0 is the faster-migrating band, and in our experiments this band was localized at approximately 38 kDa (Fig. 1A). Cx43-P1 and -P2 are the two slower-migrating bands and were localized at 41 and 43 kDa, respectively. The P2 band often appeared as a double band. Cx43 also consistently displayed a weak band at 50 kDa. In quantifying the various Cx43 bands on western blots, we found the depletion of ITCH to be associated with an approximate 70% increase in the Cx43 protein level compared with that in control siRNA-transfected cells (Fig. 1A). As determined by confocal fluorescence microscopy, depletion of ITCH was associated with larger gap junctions at the plasma membrane (Fig. 1B). To obtain a quantitative assessment of the alterations in gap junction levels in response to ITCH knockdown, the Cx43 signal localized to the plasma membrane, identified by wheat germ agglutinin (WGA) staining, was measured in the confocal fluorescence microscopy images. This analysis suggested that the area of the plasma membrane containing gap junctions was approximately 2-fold higher in the images of HeLa-Cx43 cells that were depleted of ITCH as compared to control cells (Fig. 1C), whereas the counted number of detectable gap junctions was not significantly altered in response to ITCH knockdown (Fig. 1D).

Similar effects on the Cx43 protein level (Suppl. Fig. S1A,B), gap junction size (Suppl. Fig. S2A,B) and the number of gap junctions detected (Suppl. Fig. S2A,C) were observed when HeLa-Cx43 cells were singly transfected with five additional, independent siRNA sequences against ITCH (termed ITCH#2–6; ITCH#1 corresponds to the siRNA sequence used in the experiments whose results are presented in Fig. 1). Among these, siRNA sequence #3 was less efficient in increasing the Cx43 protein level as compared to the other sequences (Suppl. Fig. S1A,B). This finding is in accordance with the observation that this siRNA sequence appeared to be the least efficient in knocking down ITCH among those sequences tested (Suppl. Fig. S1C). We also confirmed that none of the six *ITCH***-**targeting siRNA sequences non-specifically depleted the cells of NEDD4 and SMURF2, two other members of the NEDD4 family that regulate the Cx43 protein level, although the siRNA sequences #2 and #6 were found to cause an approximate 30% reduction in the NEDD4 protein level (Suppl. Fig. S1A,C) [35, 47, 50].

Next, we examined whether ITCH regulates the Cx43 protein level and gap junction size in cervical carcinoma cells expressing Cx43 endogenously. For this purpose, we used C33A cells, which express Cx43 at relatively high levels and form functional gap junctions [47, 65]. As determined with quantitative western blotting, an approximate 115% rise in the Cx43 protein level was observed after transfecting these cells with siRNA against *ITCH* for 96 h in comparison to the level in control-siRNA-transfected cells (Fig. 1E). Confocal fluorescence microscopy analyses indicated that the enhanced cellular level of Cx43 protein under these conditions was associated with both an increased size of Cx43-based gap junctions (Fig. 1F and G) as well as an increased number of gap junctions (Fig. 1F and H).

### ITCH controls gap junction intercellular communication

**Fig. 2:**
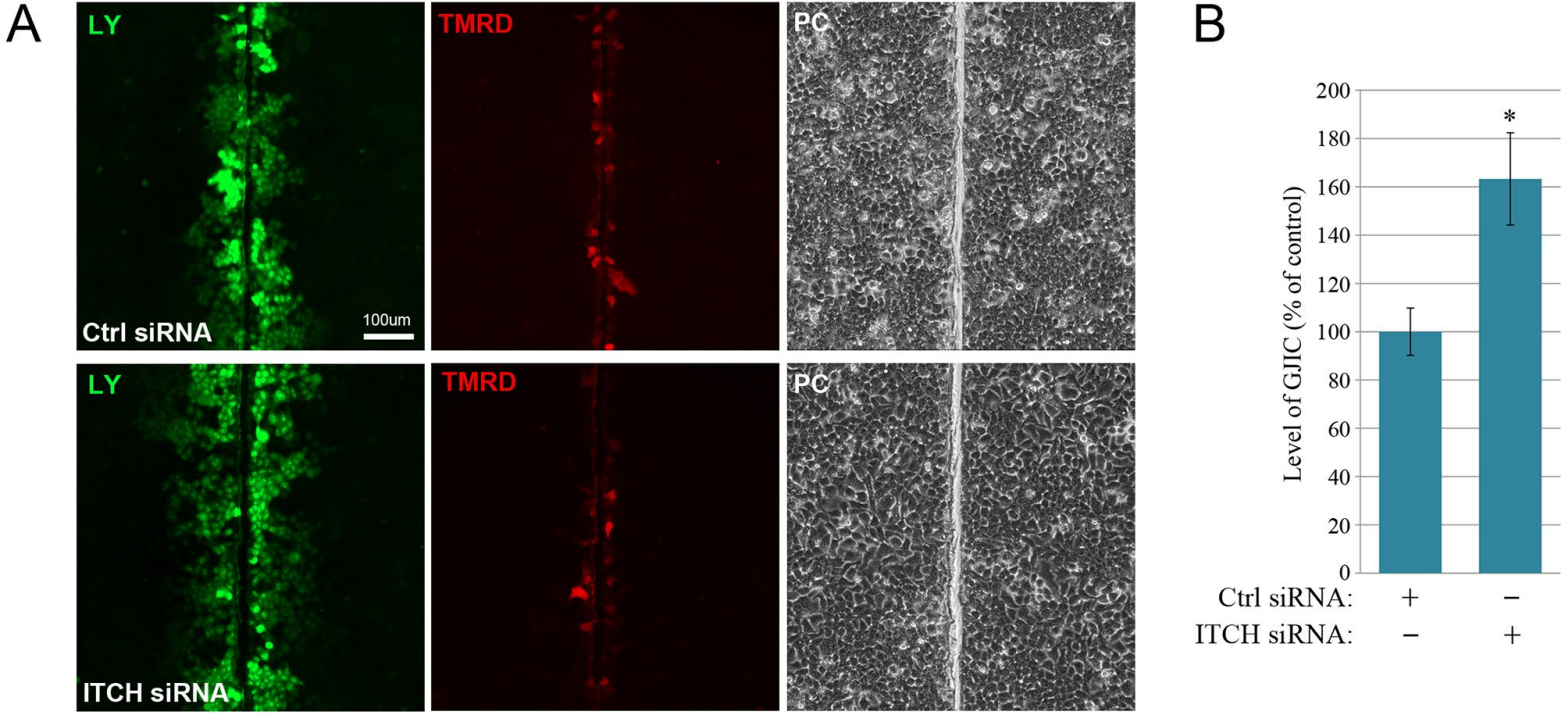
Role of ITCH in regulating gap junction intercellular communication in HeLa-Cx43 cells. (**A**) HeLa-Cx43 cells were transfected with control siRNA or with an siRNA sequence against ITCH (corresponding to siRNA #1 in Suppl. Fig. S1 A,B), as indicated, for 96 h. Gap junction intercellular communication was assessed by performing a scrape loading-dye transfer assay. Scale bar, 100 μm, applies to all images. LY, Lucifer Yellow; TMRD, tetramethylrhodamine-conjugated Dextran; PC, phase contrast. (**B**) Quantification of gap junction intercellular communication by performing the scrape loading-dye transfer assay experiment shown in A. Values shown are the means ± S.E.M. of four independent experiments. *p < 0.0005

We next investigated whether the increased size of gap junctions in response to ITCH knockdown is associated with elevated gap junctional intercellular communication. In these experiments, gap junction intercellular communication was investigated by performing a quantitative scrape loading-dye transfer assay [67]. This analysis revealed that depletion of ITCH in HeLa-Cx43 cells is associated with an increase in gap junctional intercellular communication of approximately 75% compared with that in control-siRNA-transfected cells (Fig. 2A,B).

### ITCH interacts with Cx43 and regulates its ubiquitination level

**Fig. 3:**
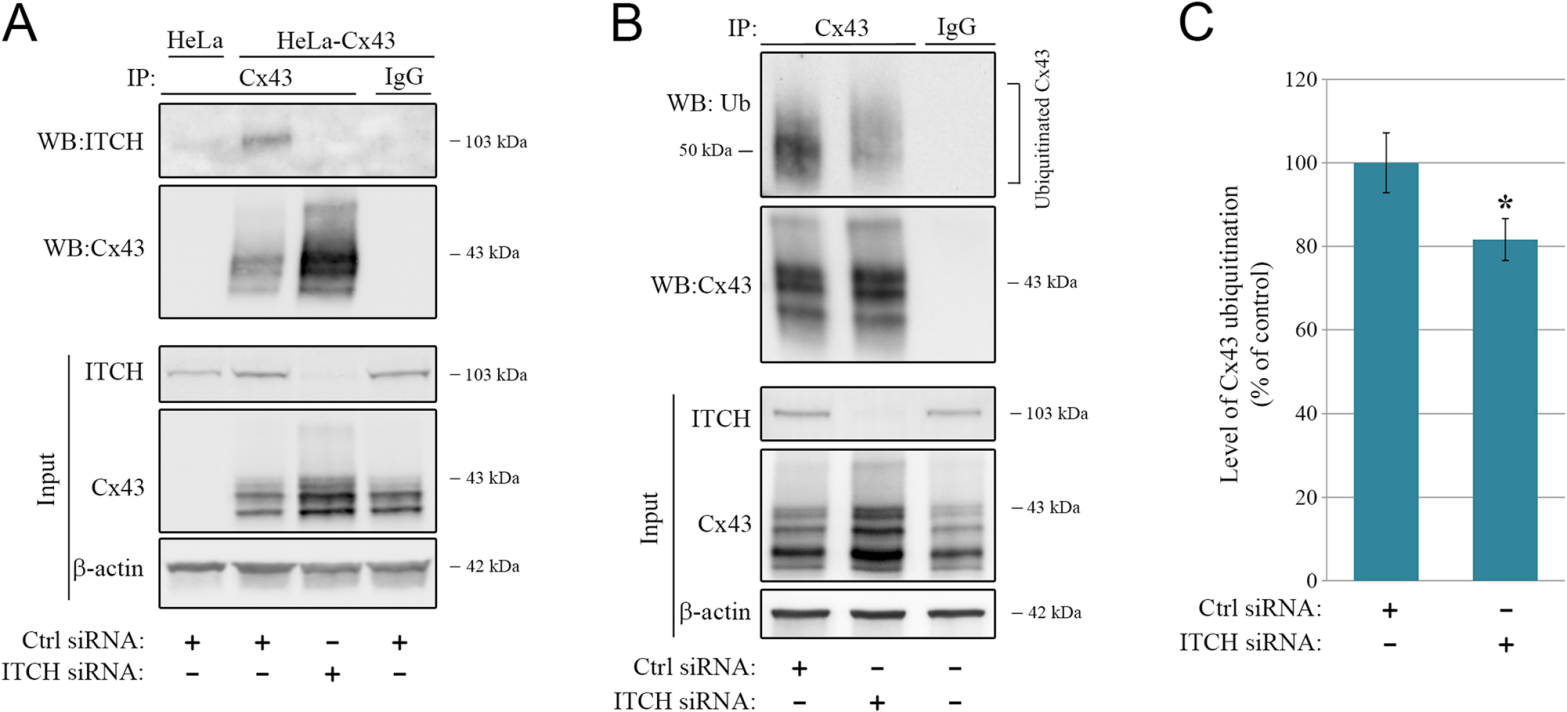
ITCH interacts with Cx43 and regulates its ubiquitination status. (**A**) HeLa-Cx43 cells were transfected with control siRNA or with an siRNA sequence against ITCH, as indicated, for 96 h. Cell lysates were then subjected to immunoprecipitation with anti-Cx43 antibodies or with rabbit IgG as negative control. HeLa cells that do not express Cx43 were included as a negative control. Equal amounts of immunoprecipitates were subjected to SDS-PAGE. Co-immunoprecipitated ITCH was detected with western blotting by using anti-ITCH antibodies (upper panel) and immunoprecipitated Cx43 was detected by using anti-Cx43 antibodies (lower panel). Also shown is the relative expression of Cx43, ITCH, and β-actin in cell lysates prior to immunoprecipitation (Input). Molecular mass in kDa is indicated. (**B**) HeLa-Cx43 cells were transfected with control siRNA or with an siRNA sequence against ITCH (corresponding to siRNA #1 in Suppl. Figure 1 A,B), as indicated, for 96 h. Cell lysates were then subjected to immunoprecipitation by using anti-Cx43 antibodies or with rabbit IgG as a negative control, and equal amounts of immunoprecipitates were subjected to SDS-PAGE. Ubiquitinated Cx43 was detected with western blotting by using anti-ubiquitin antibodies (upper panel). The blot was stripped and reprobed with anti-Cx43 antibodies (lower panel). Also shown is the relative expression of Cx43, ITCH, and β-actin in cell lysates prior to immunoprecipitation (Input). Molecular mass in kDa is indicated. (**C**) Quantification of ubiquitinated Cx43 based on the data obtained in B. For each lane, the level of ubiquitin immunoreactivity was normalized to the level of Cx43 immunoreactivity in the immunoprecipitates. Values shown are the means ± S.E.M. of 12 independent experiments. *p < 0.05

Next, we investigated whether Cx43 interacts with ITCH, as determined by co-immunoprecipitation. To this end, HeLa-Cx43 cells were transfected with siRNA targeting *ITCH* or with control siRNA for 96 h, followed by immunoprecipitation of Cx43 by using an anti-Cx43 antibody. As determined by western blotting, ITCH was found to co-immunoprecipitate with Cx43 in HeLa-Cx43 cells transfected with control-siRNA but not in cells depleted of ITCH (Fig. 3A). These data indicate that ITCH interacts with Cx43, either directly or indirectly via another protein.

To determine how ITCH depletion affects the Cx43 ubiquitination status, we subjected HeLa-Cx43 cells transfected with siRNA targeting ITCH or with control siRNA for 96 h to immunoprecipitation by using an anti-Cx43 antibody. The Cx43 ubiquitination level in the control and ITCH-depleted cells was then compared by performing western blotting of the immunoprecipitates by using anti-ubiquitin antibodies. The ubiquitinated Cx43 in HeLa-Cx43 cells displayed a smear with the highest intensity at approximately 50 kDa, in accordance with the results of previous studies (Fig. 3B) [47]. A quantitative analysis of the ubiquitin signals on western blots indicated that the depletion of ITCH with the same siRNA sequence targeting ITCH as described earlier (corresponding to ITCH #1 in Suppl. Fig. S1) was associated with an approximate 20% reduction in the Cx43 ubiquitination level (Fig. 3C). Depletion of ITCH by using another siRNA sequence targeting ITCH (corresponding to ITCH #2 in Suppl. Fig. S1) resulted in an approximate 35% reduction in Cx43 ubiquitination (Suppl. Fig. S3A,B). Collectively, these data indicate that ITCH regulates Cx43 ubiquitination in HeLa-Cx43 cells.

### ITCH promotes the loss of Cx43-based gap junctions in a process that requires an intact HECT domain

**Fig. 4:**
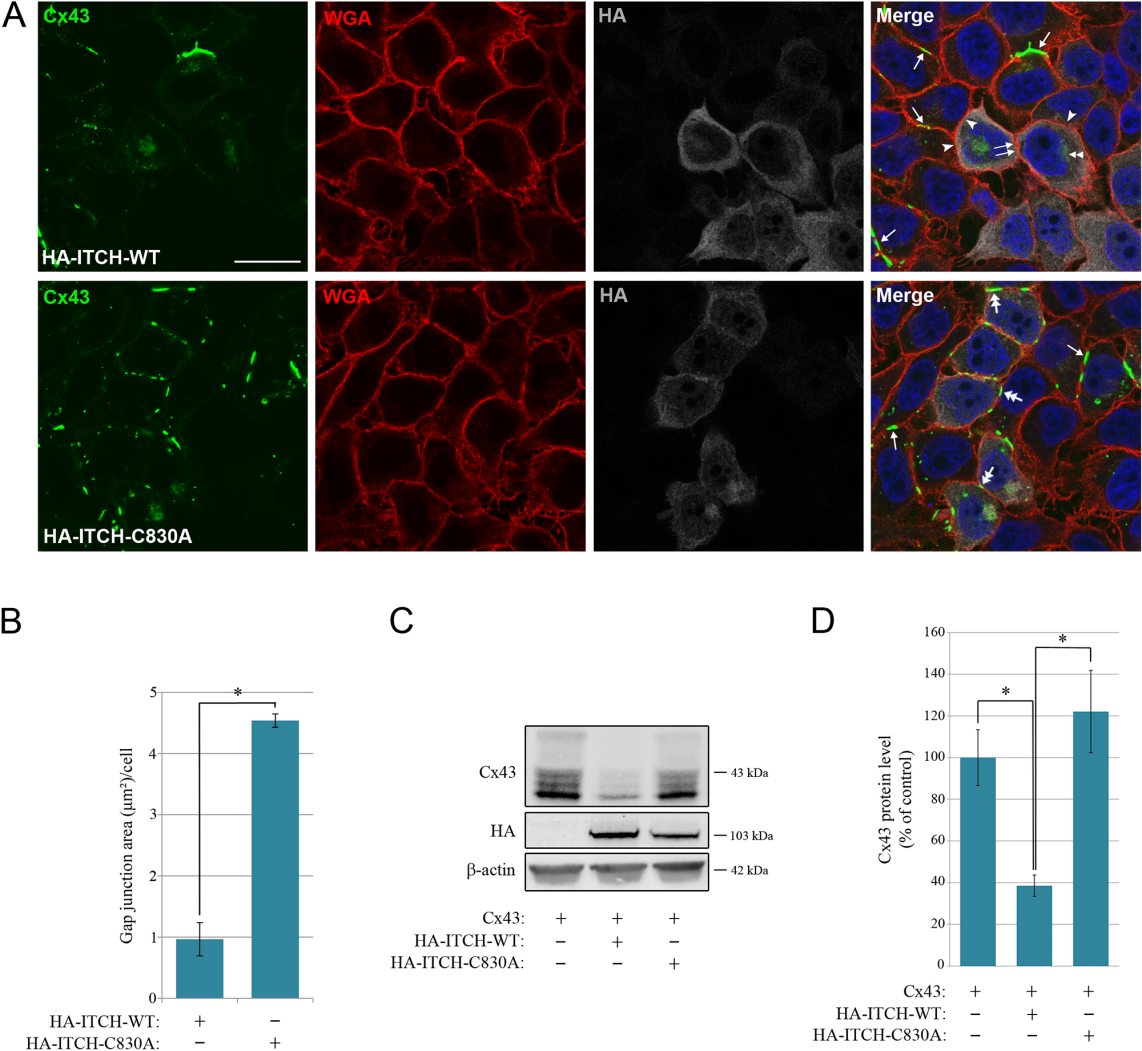
Effect of ectopic expression of ITCH on the level of Cx43-based gap junctions and the cellular Cx43 protein level. (**A**) HeLa-Cx43 cells were transfected with HA-ITCH-WT or HA-ITCH-C830A for 48 h, as indicated. The cells were fixed and stained with anti-Cx43 (green) and anti-HA (grey) antibodies followed by Alexa488- and Alexa647-conjugated secondary antibodies. The plasma membrane was stained with Alexa555-conjugated WGA (red). Nuclei were stained with Hoechst 33342 (blue). The cells were visualized by confocal fluorescence microscopy. Scale bar, 10 μm, applies for all images. Arrows indicate examples of Cx43-based gap junctions formed between adjacent cells that do not express HA-ITCH-WT or HA-ITCH-C830A. Paired arrows indicate an example of a region of the plasma membrane between two adjacent cells that both express HA-ITCH-WT, where Cx43-based gap junctions are not formed. Arrowheads indicate examples of regions of the plasma membrane between two adjacent cells, one of which express HA-ITCH-WT and the other not, where Cx43-based gap junctions are not formed. Two-headed arrows indicate examples of Cx43-based gap junctions formed in the plasma membrane of cells expressing HA-ITCH-C830A. Double arrowhead indicate an example of Cx43 staining in vesicular structures localized in the perinuclear area that does not appear to be affected by ectopic overexpression of HA-ITCH-WT. (**B**) Quantification of the area of Cx43-based gap junctions per cell based on confocal fluorescence microscopy images in A. Values shown are the means ± S.E.M. of three independent experiments. *p < 0.0005. (**C**) HeLa cells negative for Cx43 were co-transfected with Cx43 and empty vector, Cx43 and HA-ITCH-WT, or Cx43 and HA-ITCH-C830A, as indicated, for 48 h. Cell lysates were then prepared and equal amounts of total cell protein were subjected to SDS-PAGE. Cx43 and β-actin were detected by western blotting. Molecular mass in kDa is indicated. (**D**) The intensities of the Cx43 bands on western blots shown in C were quantified and normalized to the level of β-actin. Values shown are the means ± S.E.M. of three independent experiments. *p < 0.05

We next investigated the effect of ectopic overexpression of ITCH on gap junction levels in HeLa-Cx43 cells. Notably, transfection of HA-ITCH-wild type (HA-ITCH-WT) was found to cause a nearly complete loss of Cx43-based gap junctions, as determined by confocal fluorescence microscopy (Fig. 4A). Such a loss was observed between adjacent cells that were both transfected with HA-ITCH-WT, as well as between adjacent cells of which only one was transfected with HA-ITCH-WT. Moreover, cells ectopically overexpressing HA-ITCH-WT displayed low Cx43 staining in intracellular vesicular structures, except for some Cx43 staining in vesicular structures localized in the perinuclear area. To investigate the role of the HECT domain in ITCH-induced loss of Cx43-based gap junctions, we transfected the cells with HA-ITCH-C830A, which is catalytically inactive due to a point mutation in the catalytic cysteine of the HECT domain. In contrast to that of HA-ITCH-WT, ectopic overexpression of HA-ITCH-C830A did not have any apparent effect on gap junction levels (Fig. 4A). Quantification of the Cx43 signal in confocal fluorescence microscopy images indicated that the area of gap junctions was approximately 4.5-fold higher in HeLa-Cx43 cells that were transfected with HA-ITCH-C830A as compared to those transfected with HA-ITCH-WT (Fig. 4B).

To examine how overexpression of ITCH affects the Cx43 protein level, we transfected Cx43-negative HeLa cells with Cx43 alone or with Cx43 in combination with HA-ITCH-WT or HA-ITCH-C830A. Co-transfection with Cx43 and HA-ITCH-WT resulted in strongly reduced Cx43 protein levels compared with those for Cx43 alone (Fig. 4C). Quantification of the Cx43 signal on western blots indicated that the Cx43 protein level in cells co-transfected with Cx43 and HA-ITCH-WT was approximately 40% of that in cells transfected with Cx43 only (Fig. 4D). In contrast, cells co-transfected with Cx43 and HA-ITCH-C830A had a Cx43 protein level that was comparable to that in cells transfected with Cx43 alone (Fig. 4D). Together, these results suggest that ectopic overexpression of ITCH in HeLa-Cx43 cells is associated with loss of gap junctions and reduced levels of Cx43 protein and that both processes depend on a functional HECT domain.

### ITCH-induced loss of Cx43 is due to increased lysosomal degradation

**Fig. 5:**
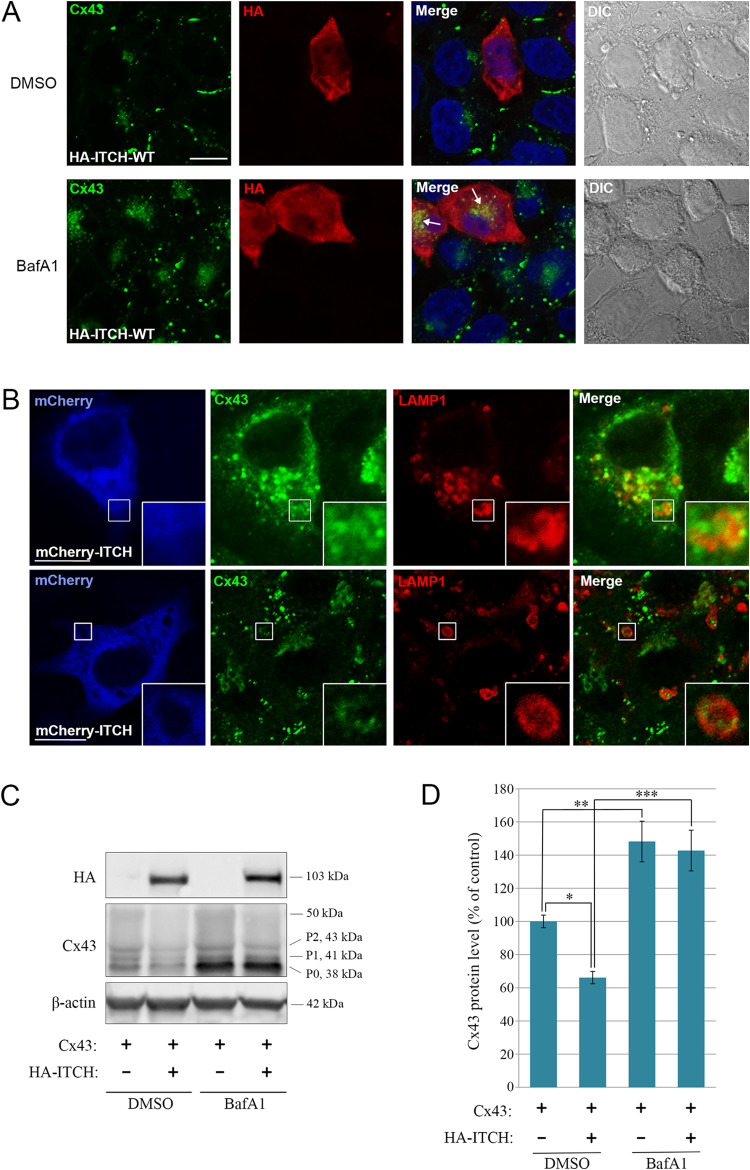
Effect of bafilomycin A1 on the ITCH-induced loss of Cx43 protein. (**A**) C33A cells were transfected with HA-ITCH-WT for 48 h. The cells were treated with DMSO (vector) or bafilomycin A1 (BafA1; 200 nM) for the last 18 h of the transfection. The cells were fixed and stained with anti-Cx43 (green) and anti-HA (red) antibodies followed by Alexa488- and Alexa594-conjugated secondary antibodies. Nuclei were stained with Hoechst 33342 (blue). The cells were visualized by confocal fluorescence microscopy. Scale bars, 10 μm. (**B**) C33A cells were transfected with mCherry-ITCH-WT for 48 h. The cells were treated with bafilomycin A1 for 18 h (upper panel) or 5 h (lower panel). Cx43 (green) and LAMP1 (red) were visualized by confocal fluorescence microscopy, as indicated. Scale bars, 10 μm. LAMP1 was stained by using Alexa405-conjugated secondary antibodies, and, following image capture, the LAMP1 signal was converted to red for better visualization of possible colocalization between LAMP1 and Cx43, and the mCherry-ITCH signal was converted to blue. (**C**) HeLa cells negative for Cx43 were co-transfected with Cx43 and empty vector or with Cx43 and HA-ITCH-WT, as indicated, for 48 h. For the last 18 h of the transfection, the cells were treated with DMSO (vector) or bafilomycin A1 (BafA1; 200 nM). Cell lysates were then prepared and equal amounts of total cell protein were subjected to SDS-PAGE. Cx43 and β-actin were detected by western blotting. Molecular mass in kDa is indicated. (**D**) The intensities of the Cx43 bands on western blots shown in C were quantified and normalized to the level of β-actin. Values shown are the means ± S.E.M. of four independent experiments. *p < 0.05, **p < 0.005, ***p < 0.001

To investigate whether ITCH-induced loss of Cx43 is due to increased degradation in lysosomes, we analyzed how this loss was affected by treatment of the cells with two lysosomal inhibitors with different mechanisms of action, bafilomycin A1 and chloroquine. As determined by confocal fluorescence microscopy, both bafilomycin A1 (Fig. 5A) and chloroquine (Suppl. Fig. S4A) strongly counteracted the HA-ITCH-induced loss of Cx43 and instead caused accumulation of Cx43 in intracellular vesicles in C33A cells expressing HA-ITCH. Both inhibitors also caused accumulation of Cx43 in intracellular vesicles in the cells within the cell monolayer that were not transfected with HA-ITCH, which is expected since previous studies have shown that the basal degradation of Cx43 occurs in lysosomes [17] (Fig. 5A and Suppl. Fig. S4A). Treatment with chloroquine, and to some extent bafilomycin A1, was associated with loss of Cx43-based gap junctions between the cells within the monolayer that were not transfected with HA-ITCH, which may be due to interruption of the recycling of Cx43 from early endosomes to the plasma membrane under these conditions. In a similar manner as in C33A cells, we found that lysosomal inhibitors counteracted the HA-ITCH-induced loss of Cx43 in HeLa-Cx43 cells, and that Cx43 under these conditions accumulated in intracellular vesicular compartments, as shown for bafilomycin A1-treated cells in Suppl. Fig. S4B.

We next aimed to characterize the types of vesicles in which Cx43 accumulated in the ITCH-transfected cells following inhibition of lysosomal activity. To this end, C33A cells transfected with mCherry-ITCH and treated with bafilomycin A1 were co-stained against Cx43 and LAMP1, a marker for late endosomes and lysosomes, and analyzed by confocal fluorescence microscopy. Cx43 was under these conditions often found to partly colocalize with LAMP1 in vesicular structures or appeared to locate inside or within the limiting membrane of large, LAMP1-positive vesicular structures (Fig. 5B).

To further investigate the effect of lysosomal inhibition on the ability of ITCH to promote degradation of Cx43, HeLa cells negative for Cx43 were transfected with only Cx43 or with Cx43 in combination with HA-ITCH and then treated with bafilomycin A1 for 18 h. Bafilomycin A1 treatment resulted in a considerable increase in the level of Cx43 protein in cells transfected with Cx43 alone, which is expected since the basal degradation of Cx43 in lysosomes is inhibited under these conditions (Fig. 5C) [17]. Quantification of the Cx43 signal indicated that the cellular level of Cx43 was approximately 50% higher under these conditions (Fig. 5D). Notably, in accordance with the confocal fluorescence microscopy results, bafilomycin A1 completely counteracted the ability of HA-ITCH to induce loss of Cx43 protein (Fig. 5C,D). As determined by confocal fluorescence microscopy, Cx43 was under these conditions found to localize in intracellular vesicular compartments (Suppl. Fig. S4C), in accordance with the observations made in C33A (Fig. 5A,B) and HeLa-Cx43 cells (Suppl. Fig. S4B). Similar results were obtained when the cells were treated with chloroquine (Suppl. Fig. S4C). Treatment of HeLa cells with bafilomycin A1 or chloroquine was not associated with significant changes in the level of endogenous ITCH (Suppl. Fig. S4D,E). In sum, these observations suggest that ITCH promotes the sorting of Cx43 to lysosomes, resulting in loss of gap junctions.

### The interaction between ITCH and Cx43 involves a PY motif in the Cx43 C-terminal tail

**Fig. 6:**
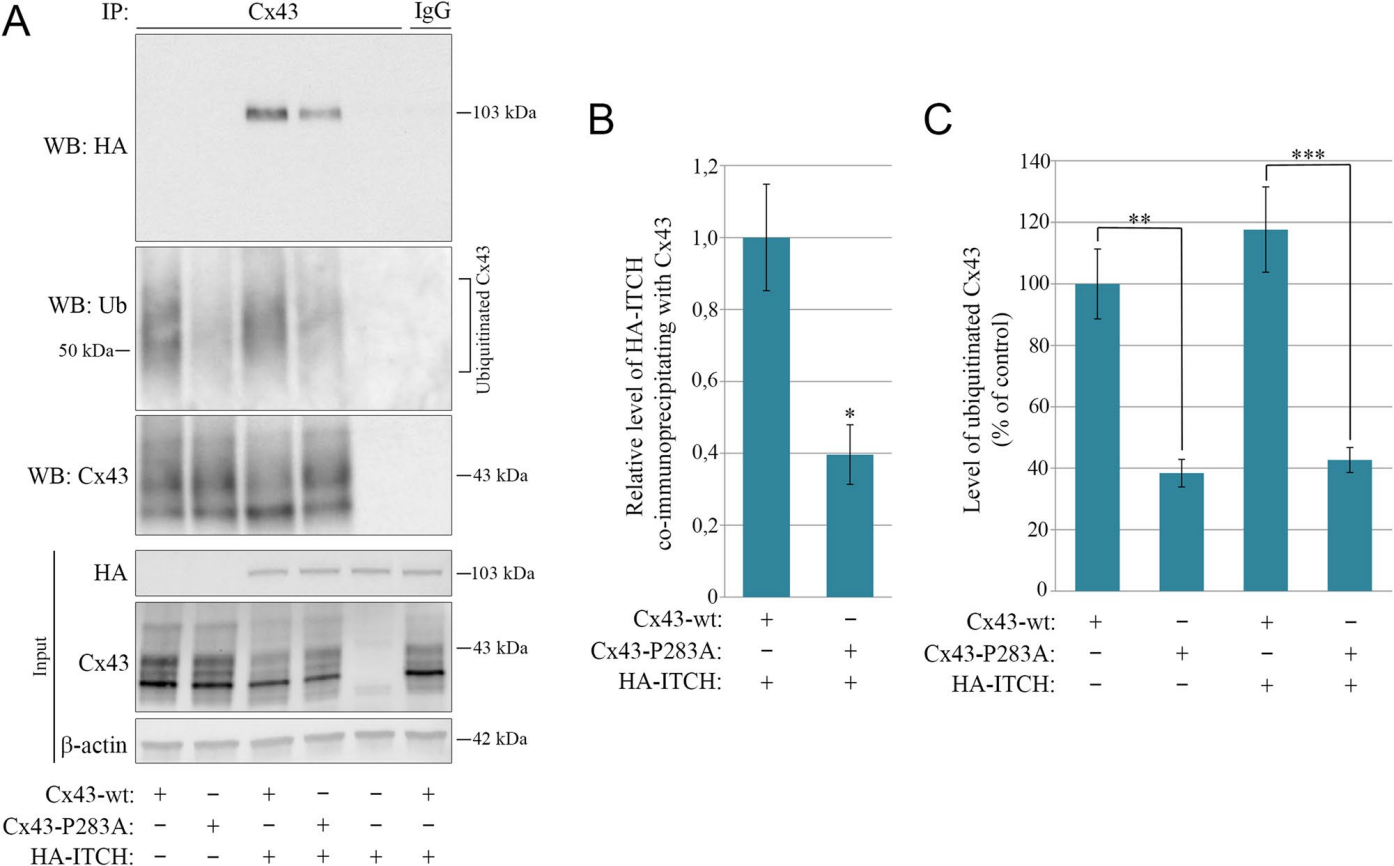
Effect of mutating the PY motif on the interaction between ITCH and Cx43 and on Cx43 ubiquitination. (**A**) HeLa cells negative for Cx43 were co-transfected with with either Cx43-WT or Cx43-P283A and HA-ITCH-WT or empty plasmid, as indicated, for 48 h. Cell lysates were then subjected to immunoprecipitation with anti-Cx43 antibodies or with rabbit IgG as control, and equal amounts of immunoprecipitates were subjected to SDS-PAGE. HA-ITCH-WT in the immunoprecipitates was detected with western blotting by using anti-HA antibodies and ubiquitinated Cx43 was detected by using anti-ubiquitin antibodies, as indicated (two upper panels, respectively). The blot was stripped and reprobed with anti-Cx43 antibodies, as indicated (lower panel). Also shown is the relative expression of Cx43, HA-ITCH-WT, and β-actin in cell lysates prior to immunoprecipitation (Input). Molecular mass in kDa is indicated. (**B**) Quantification of the relative level of HA-ITCH that co-immunoprecipitates with Cx43-WT and Cx43-P283A based on the data obtained in A. For each lane, the level of HA-ITCH in the immunoprecipitate was normalized to the level of Cx43 in the immunoprecipitate. Values shown are the means ± S.E.M. of four independent experiments. *p < 0.05. (**C**) Quantification of the relative level of Cx43 ubiquitination in the various samples based on the data obtained in A. For each lane, the level of ubiquitin immunoreactivity in the immunoprecipitate was normalized to the level of Cx43 in the immunoprecipitate. Values shown are the means ± S.E.M. of four independent experiments. *p < 0.05, **p < 0.005, ***p < 0.001

ITCH contains four class 1 WW domains, which are protein modules that mediate interactions between proteins through binding to PY motifs (PPXY, where P is proline, X is any amino acid, and Y is tyrosine) [68]. A PY motif is located in the C-terminal tail of Cx43 (^283^PPGY^286^) [69]. To determine whether the interaction between Cx43 and ITCH involves this motif, we asked whether a substitution of the first proline of the motif by an alanine (Cx43-P283A) affects the interaction. To this end, HeLa cells that do not express Cx43 endogenously were transfected with Cx43-WT or Cx43-P283A either alone or in combination with HA-ITCH-WT. Cx43 was then immunoprecipitated and the effect of mutating the PY motif on the ability of Cx43 to co-immunoprecipitate HA-ITCH-WT was determined by western blotting. The data suggested that HA-ITCH-WT co-precipitates considerably less efficiently with Cx43-P283A than it does with Cx43-WT (Fig. 6A). Quantification of the western blot results suggested that the level of HA-ITCH-WT that co-precipitated with Cx43 was reduced by approximately 60% upon the mutation of the PY motif (Fig. 6B). As expected, mutation of the PY motif was associated with a decrease in the Cx43 ubiquitination level (Fig. 6C). These results suggest that the interaction between ITCH and Cx43 involves the PY motif.

### The ability of ITCH to promote loss of Cx43-based gap junctions and degradation of Cx43 is dependent on an intact PY motif

**Fig. 7:**
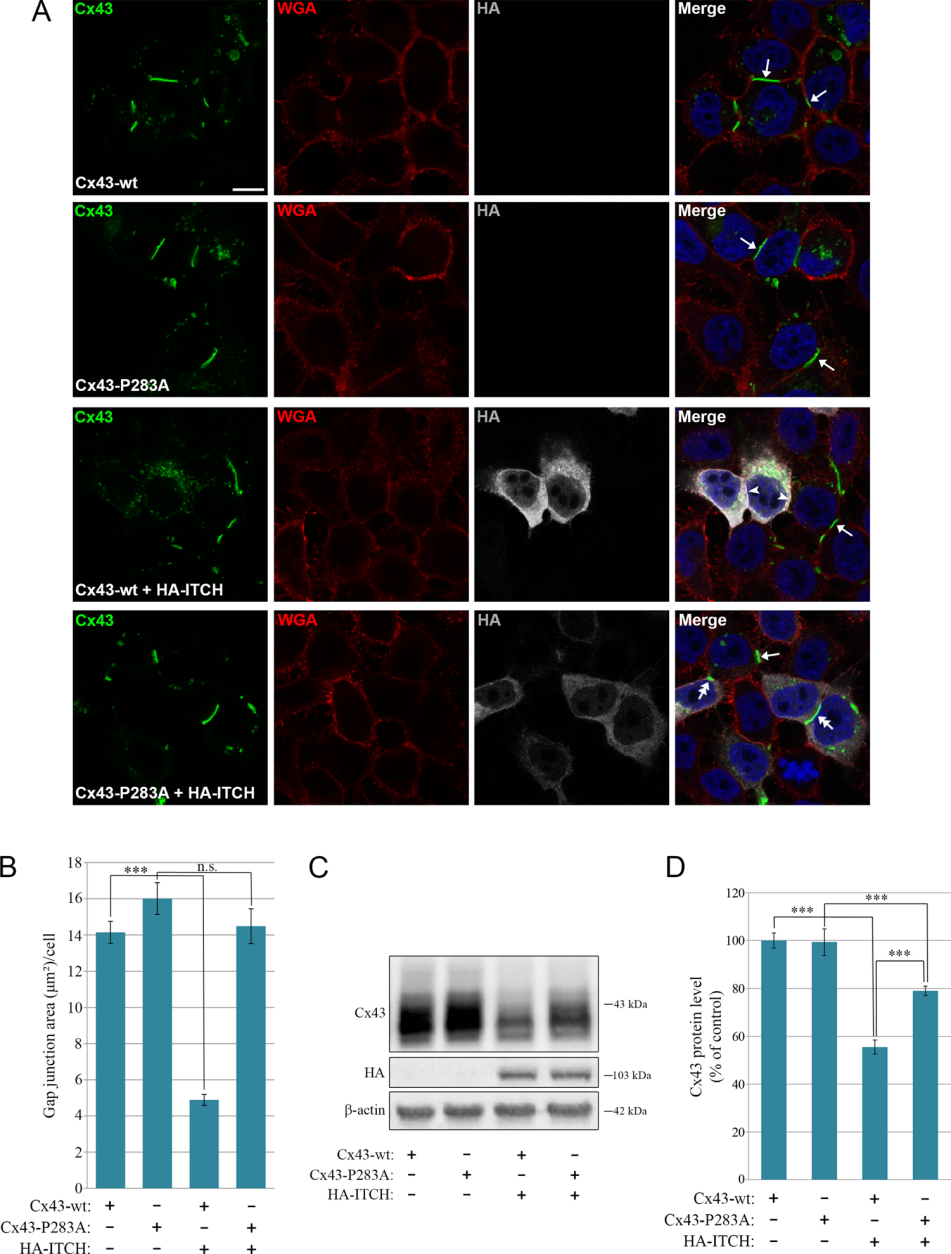
Effect of mutating the PY motif on the ability of ITCH to promote loss of gap junctions and degradation of Cx43. HeLa cells negative for Cx43 were co-transfected with either Cx43-WT or Cx43-P283A and empty plasmid, Cx43-WT and HA-ITCH, or Cx43-P283A and HA-ITCH, as indicated, for 48 h. (**A**) The cells were fixed and stained with anti-Cx43 (green) and anti-HA (grey) antibodies followed by Alexa488- and Alexa647-conjugated secondary antibodies. The plasma membrane was stained with Alexa555-conjugated WGA (red). Nuclei were stained with Hoechst 33342 (blue). The cells were visualized by confocal fluorescence microscopy. Scale bars, 10 μm. Arrows indicate examples of Cx43-based gap junctions formed between adjacent cells expressing only Cx43-WT or Cx43-P283A and not HA-ITCH-WT. Arrowheads indicate examples of regions of the plasma membrane of cells co-expressing Cx43-WT and HA-ITCH-WT, where Cx43-based gap junctions are not formed. Two-headed arrows indicate examples of Cx43-based gap junctions formed in the plasma membrane of cells co-expressing Cx43-P283A and HA-ITCH-WT. (**B**) Quantification of the area of Cx43-based gap junctions per cell based on confocal fluorescence microscopy images in A. Values shown are the means ± S.E.M. of three independent experiments. *p < 0.001. n.s., not significant. (**C**) Cell lysates were prepared and equal amounts of total cell protein were subjected to SDS-PAGE. Cx43 and β-actin were detected by western blotting. Molecular mass in kDa is indicated. **(D)** The intensities of the Cx43 bands on western blots shown in C were quantified and normalized to the level of β-actin. Values shown are the means ± S.E.M. of six independent experiments. ***p < 0.001

To investigate the functional implications of the finding that the interaction between ITCH and Cx43 involves the PY motif, we asked whether the ability of ITCH to induce loss of Cx43-based gap junctions is dependent on an intact PY motif. In these experiments, HeLa cells deficient in Cx43 were transiently transfected with Cx43-WT or Cx43-P283A either alone or in combination with HA-ITCH-WT, and the ability of the cells to form gap junctions was determined by confocal fluorescence microscopy. The HeLa cells transfected with either Cx43-WT or Cx43-P283A alone were observed to form gap junctions, as expected (Fig. 7A). In line with the data obtained on HeLa-Cx43 cells (Fig. 4A), when HeLa cells deficient in Cx43 were co-transfected with Cx43-WT and HA-ITCH-WT, no gap junctions were observed (Fig. 7A). In general, there was low intracellular staining of Cx43 under these conditions, except for some weak Cx43 staining in intracellular vesicular compartments. In contrast, when HeLa cells deficient in Cx43 were co-transfected with Cx43-P283A and HA-ITCH-WT, gap junctions were formed in a manner that was similar to that in cells transfected with Cx43 or Cx43-P283A alone (Fig. 7A). Quantification of the Cx43 signal in confocal fluorescence microscopy images suggested that there was a statistically significant loss of Cx43-based gap junctions when HA-ITCH-WT was co-transfected with Cx43-wt, but not with Cx43-P283A (Fig. 7B).

We also investigated how mutation of the PY motif affected the ability of ITCH to promote degradation of Cx43, as assessed by western blotting. HeLa cells that do not express Cx43 endogenously that were transiently transfected with Cx43-P283A displayed a similar Cx43 protein level to that in cells transfected with Cx43-WT (Fig. 7C,D). In agreement with the data obtained earlier (Fig. 4C,D), when HeLa cells that do not express Cx43 endogenously were co-transfected with Cx43-WT and HA-ITCH-WT, significantly lower levels of Cx43 were observed compared with those in cells transfected with Cx43 alone (Fig. 7C,D). In contrast, HA-ITCH was considerably less efficient in inducing degradation of Cx43-P283A (Fig. 7C,D). Quantification of the Cx43 signal indicated that the ability of HA-ITCH to induce Cx43 degradation was counteracted in a statistically significant manner when the PY motif was mutated (Fig. 7C,D). Taken together, these observations indicate that the ability of ITCH to induce degradation of Cx43 is dependent on the PY motif in the C-terminal tail of Cx43.

### The interaction between ITCH and Cx43 is strongly increased upon lysosomal inhibition

**Fig. 8:**
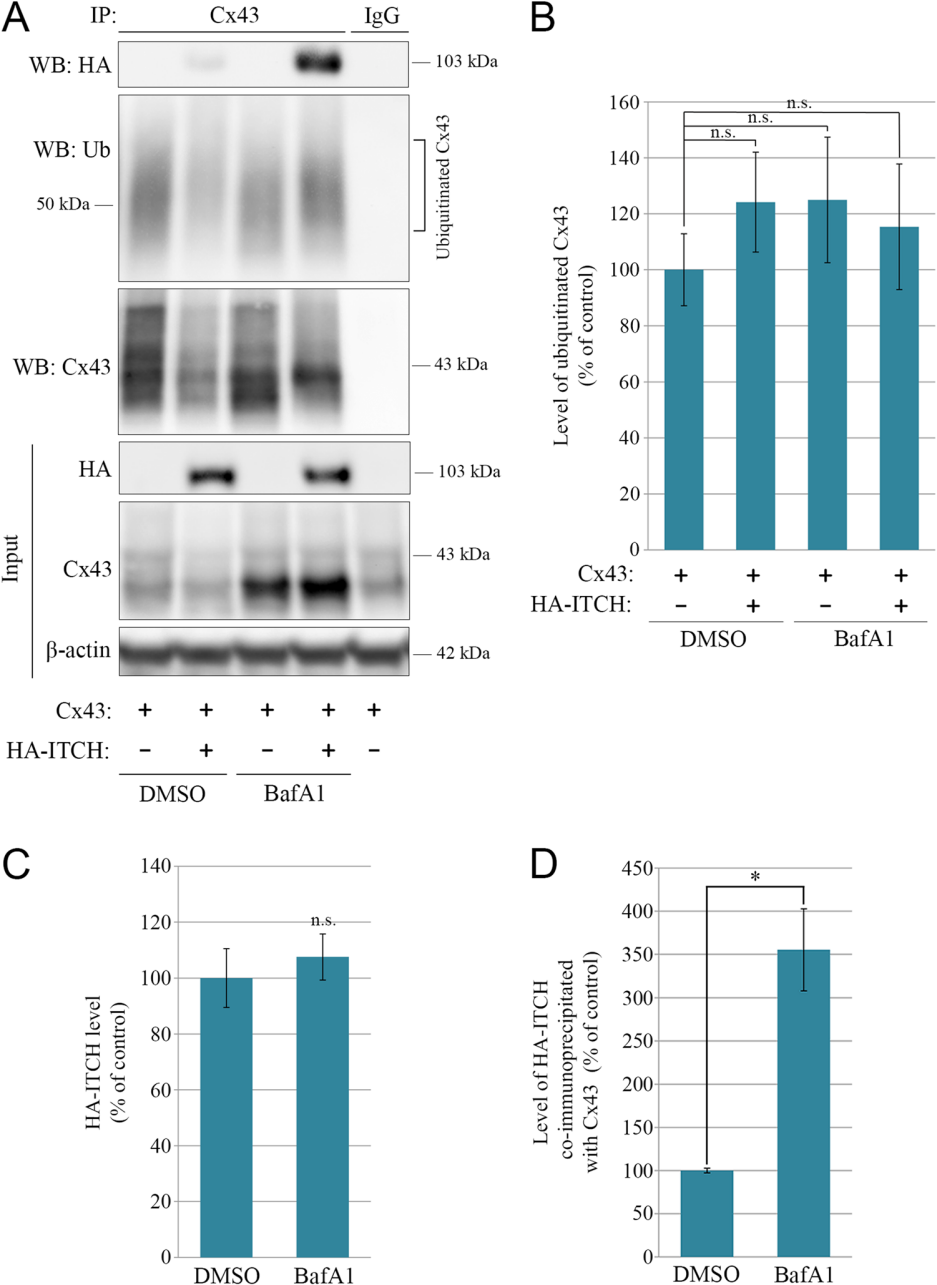
Effect of bafilomycin A1 on the interaction between ITCH and Cx43. (**A**) HeLa cells negative for Cx43 were co-transfected with Cx43 and empty vector or with Cx43 and HA-ITCH, as indicated, for 48 h. For the last 18 h of the transfection, the cells were treated with DMSO (vector) or bafilomycin A1 (BafA1; 200 nM), as indicated. Cell lysates were then subjected to immunoprecipitation with anti-Cx43 antibodies or with rabbit IgG as negative control, and equal amounts of the immunoprecipitates were subjected to SDS-PAGE. HA-ITCH in the immunoprecipitates was detected with western blotting by using anti-HA antibodies and ubiquitinated Cx43 was detected by using anti-ubiquitin antibodies, as indicated (two upper panels, respectively). The blot was stripped and reprobed with anti-Cx43 antibodies, as indicated (lower panel). Also shown is the relative expression of Cx43, HA-ITCH, and β-actin in cell lysates prior to immunoprecipitation (Input). Molecular mass in kDa is indicated. (**B**) Quantification of the relative level of Cx43 ubiquitination in the various samples based on the data obtained in A. For each lane, the level of ubiquitin immunoreactivity in the immunoprecipitate was normalized to the level of Cx43 in the immunoprecipitate. Values shown are the means ± S.E.M. of five independent experiments. n.s., not significant. (**C**) Quantification of the relative level of HA-ITCH in the Input samples shown in A. For each lane, the level of HA-ITCH in the lysates was normalized to the level of β-actin. Values shown are the means ± S.E.M. of five independent experiments. n.s., not significant. (**D**) Quantification of the relative level of HA-ITCH that co-immunoprecipitated with Cx43 based on the data obtained in A. For each lane, the level of HA-ITCH in the immunoprecipitate was normalized to the level of Cx43 in the immunoprecipitate. Values shown are the means ± S.E.M. of five independent experiments. *p < 0.05

We next investigated how the inhibition of the ITCH-induced degradation of Cx43 in response to bafilomycin A1 treatment influences the Cx43 ubiquitination status and the interaction between ITCH and Cx43 in HeLa cells. It has previously been shown that Cx43 is deubiquitinated prior to its degradation in lysosomes, [37, 40]. In agreement with the results of these studies, in the present study, bafilomycin A1 treatment was not associated with an increase in Cx43 ubiquitination either in cells transfected with Cx43 alone or in cells co-transfected with Cx43 and HA-ITCH (Fig. 8A,B). However, interestingly, although the total cellular level of HA-ITCH did not increase in response to bafilomycin A1 treatment (Fig. 8A,C), there was an almost four-fold increase in the level of HA-ITCH that co-immunoprecipitated with Cx43 under these conditions (Fig. 8A,D). These results suggest that in HeLa cells, the counteraction of the lysosomal degradation of Cx43 in response to bafilomycin A1 treatment is associated with increased interaction between ITCH and Cx43, but this is not associated with enhanced Cx43 ubiquitination.

## Discussion

Herein, we have identified ITCH as a novel regulator of gap junction intercellular communication. Our study suggests that ITCH negatively controls the size of gap junctions in cervical cancer cells by promoting the lysosomal degradation of Cx43 and thereby reducing the cellular pool of Cx43 available to form gap junctions.

The C-terminal tail of Cx43 contains several domains involved in mediating protein-protein interactions [70]. The present study adds ITCH to the list of proteins that can interact with the C-terminal tail of Cx43. In addition to the HECT domain, ITCH displays four WW domains in its central region and a C2 domain in its N-terminal region. The WW domains mediate protein-protein interactions and consist of 38–40 amino acids that include two highly conserved tryptophan residues [68]. The WW domains are divided into five classes, and those of ITCH pertain to class I, which are characterized by binding to PY motifs in other proteins [68]. NEDD4 has previously been shown to bind to Cx43 via the PY motif located in its C-terminal tail (^283^PPGY^286^) [35, 45, 46]. The data obtained here suggest that ITCH may also interact with Cx43 via the PY motif and that this motif has to be intact in order for ITCH to efficiently induce lysosomal degradation of Cx43 and the accompanying loss of gap junctions. It remains to be determined whether ITCH may bind directly to Cx43 or whether the interaction between these two proteins involves an adaptor protein. To our knowledge, these results provide the first experimental evidence that the PY motif is strictly required in order for members of the NEDD4 family to efficiently induce degradation of Cx43.

In agreement with the finding that NEDD4 and ITCH interact with Cx43 via the PY motif, mutating the first proline of this motif to a leucine (Cx43-P283L) has previously been reported to cause reduced Cx43 ubiquitination [35]. Thomas et al. have shown that transient transfection of SKHEP1 human liver epithelial cells with a Cx43-P283L mutant results in increased cellular levels of Cx43 as compared to when they are transfected with Cx43-WT [69]. Somewhat surprisingly, introducing a point mutation in the PY motif (Cx43-P283A) did not result in increased Cx43 protein levels under basal conditions in our experiments, although the Cx43 ubiquitination level was, as expected, strongly reduced under these conditions. A possible explanation for this observation may be that in addition to the PY motif, Cx43 contains three tyrosine-based sorting signals of the type YXXФ, which also are involved in mediating the endocytosis and lysosomal degradation of Cx43 [69, 71]. It is possible that these signals may compensate for the loss of a functional PY motif and efficiently induce degradation of Cx43 in HeLa cells even though the PY motif is mutated. Thomas et al. found that, in SKHEP1 cells, mutating one of the YXXФ motifs in Cx43 had a considerably stronger effect on the steady-state level of Cx43 as compared to when the PY motif was mutated [69]. These observations suggest that the YXXФ motifs may be more important than the PY motif in regulating the degradation of Cx43 under basal conditions. It should be noted that in our western blot experiments, the Cx43 band pattern in the immunoprecipitates appeared somewhat different from that in the input samples. The reason for this is currently not known. It is also not known whether this may somehow affect the results obtained regarding how the mutation of the PY motif affects the Cx43 ubiquitination status or the ability of Cx43 to co-immunoprecipitate HA-ITCH.

It has previously been shown that NEDD4 family-interacting protein 2 (NDFIP2) stabilizes Cx43 [72]. NDFIP2 is a transmembrane protein located predominantly in the Golgi apparatus and in multivesicular endosomes [73]. It contains three PY motifs, through which it binds to members of the NEDD4 family, including ITCH, causing their activation [74]. NDFIP2 has important roles in mediating the binding of members of the NEDD4 family to protein substrates that do not display PY motifs, such as the divalent metal ion transporter DMT1 [75] and Robo receptors [76]. NDFIP2 was suggested to stabilize Cx43 by causing reduced binding between members of the NEDD4 family and Cx43 due to competitive binding to NDFIP2 [72].

siRNA-mediated depletion of ITCH in HeLa-Cx43 cells was found to result in reduced Cx43 ubiquitination. However, when HeLa cells that do not express Cx43 endogenously were co-transfected with Cx43 and ITCH, there was no statistically significant increase in the Cx43 ubiquitination level compared with that in cells transfected with Cx43 only. A possible scenario is that the ectopically expressed ITCH induces ubiquitination of Cx43 under these conditions, but that Cx43 is rapidly deubiquitinated and degraded. This would make it difficult to experimentally detect an increase in the level of Cx43 ubiquitination. It is also possible that ITCH catalyzes the ubiquitination of only one or a few lysine residues in Cx43, which could also make it difficult to detect an increase in Cx43 ubiquitination under these conditions. Based on the observations that Cx43 ubiquitination is decreased upon ITCH depletion and that the interaction between ITCH and Cx43 involves the PY motif, we consider that the most likely scenario is that ITCH promotes Cx43 degradation by catalyzing its ubiquitination. However, we cannot rule out the possibility that ITCH may indirectly regulate Cx43 degradation by catalyzing the ubiquitination of another protein rather than Cx43 itself. For instance, ITCH has previously been shown to catalyze ubiquitination of hepatocyte growth factor-regulated tyrosine kinase substrate (Hrs), a component of the endosomal sorting complex required for transport (ESCRT) [77]. The effect of the ITCH-mediated ubiquitination of Hrs is, to the best of our knowledge, currently not known. However, it could potentially have implications for Cx43 degradation, since Hrs under some conditions may be involved in mediating the sorting of Cx43 from early endosomes to lysosomes [37]. Other proteins involved in endocytosis, such as endophilin-A1 [78] and SNX9 [79], are also known ITCH substrates, but whether these proteins participate in the endocytosis of Cx43-based gap junctions is, to our knowledge, not known. An important subject for future studies will be to obtain a more detailed understanding of the molecular mechanisms involved in the ITCH-mediated regulation of Cx43 degradation.

As determined by confocal fluorescence microscopy, the ITCH-induced loss of Cx43 in C33A cells was strongly counteracted by bafilomycin A1 and chloroquine, suggesting that this loss is due to degradation of Cx43 in lysosomes. Supporting this notion, C33A cells ectopically overexpressing HA-ITCH and treated with bafilomycin A1 were often found to display partial colocalization between Cx43 and LAMP1 in intracellular vesicular compartments or localization of Cx43 in large, LAMP1-positive vesicles. As determined by western blotting, the steady-state level of Cx43 increased in response to bafilomycin A1 treatment, which is in concordance with previous studies showing that the basal degradation of Cx43 involves lysosomes [14, 17]. The observation that bafilomycin A1 counteracted the ITCH-induced loss of Cx43 protein, as determined by western blotting, is in agreement with the confocal fluorescence microscopy analyses and supports the notion that the ITCH-induced loss of Cx43 is due to increased degradation of Cx43 in lysosomes. It remains to be determined where in the cell the interaction between Cx43 and ITCH occurs. One possibility is that ITCH interacts with Cx43 assembled into gap junction plaques and promotes the internalization of gap junctions, resulting in increased number of connexosomes in the cytoplasm. This could cause enhanced turnover of Cx43 due to increased fusion between connexosomes and lysosomes, increased degradation of connexosomes by autophagy, or increased trafficking of Cx43 to lysosomes via the endolysosomal pathway. An alternative scenario is that ITCH interacts with Cx43 at the limiting membrane of endosomes and promotes the sorting of Cx43 into their lumen, thereby causing increased trafficking of Cx43 to lysosomes. This could result in reduced recycling of Cx43 from endosomes to the plasma membrane and, as a consequence, depletion of the pool of Cx43 connexons at the plasma membrane available for assembly into gap junctions. A third possibility is that ITCH interacts with Cx43 before it reaches the plasma membrane and targets it directly to lysosomes, by following a trafficking pathway from early secretory compartments to lysosomes previously described by Qin et al. [17]. It is also conceivable that the interaction between ITCH and Cx43 occurs in various cellular locations, and that ITCH promotes the lysosomal degradation of Cx43 through different pathways.

Our data indicate that the accumulation of Cx43 in intracellular vesicular compartments in response to bafilomycin A1 treatment of HeLa cells is associated with enhanced interaction between Cx43 and ITCH. A possible explanation for this may be the existence of a negative feedback mechanism, in which unusually high levels of Cx43 increases its interaction with ITCH to induce the ubiquitination and lysosomal degradation of Cx43. Despite the increased interaction with ITCH under these conditions, Cx43 ubiquitination is not augmented. A possible explanation for this observation might be that the intracellular accumulation of Cx43 upon bafilomycin A1 treatment is associated with deubiquitination of the lysine residues of Cx43 that are ubiquitinated by ITCH. Previous studies have suggested that prior to its trafficking to lysosomes for degradation, Cx43 is subjected to deubiquitination at the limiting membrane of early endosomes before it is sorted into the lumen of the endosomes in a process mediated by the deubiquitinating enzyme AMSH [37, 40]. Alternatively, it could reflect that ITCH may not directly regulate Cx43 by catalyzing its ubiquitination, but rather control Cx43 indirectly via ubiquitination of another protein, as discussed above.

Cancer development is usually associated with the loss of Cx43-based gap junctions, and Cx43 has been shown to act as a tumor suppressor in various tissue types [3–5]. The mechanisms involved in the loss of gap junctions during cancer pathogenesis are poorly understood. There is, however, increasing evidence suggesting that aberrant endocytosis and/or trafficking of Cx43 along the endolysosomal pathway may be involved in mediating the loss of Cx43-based gap junctions in cancer cells. For instance, in some human pancreatic cancer cells, connexons formed by Cx43 fail to form gap junctions because they undergo endocytosis prior to gap junction assembly and instead localize in cytoplasmic vesicular compartments, many of which are LAMP1 positive [12]. In addition, in some human glioblastoma cell lines, Cx43 predominantly localizes in late endosomes and lysosomes, and only a minor fraction of the cellular pool of Cx43 is assembled into gap junctions [24]. Moreover, colorectal cancer pathogenesis is associated with either loss of Cx43 expression or relocalization of Cx43 from the plasma membrane to intracellular compartments, including endosomes [25]. Cervical cancer progression is associated with dysregulation of the intracellular trafficking of Cx43, resulting in loss of gap junctions, but the molecular mechanisms involved in these processes are currently incompletely understood [65, 66, 80, 81]. ITCH is considered to have an important role in cancer development because of its capacity to control the ubiquitination and degradation of several proto-oncogenic proteins such as c-Jun, Cbl-b, and c-FLIP [57–59] and tumor suppressor proteins, such as p63, p73, and LATS1 [60–62]. Interestingly, upregulation of ITCH has been associated with cervical squamous cell carcinoma progression [64]. Thus, the finding that ITCH interacts with Cx43 and targets it for degradation in cervical cancer cells may be an important step toward understanding the molecular basis underlying the loss of Cx43-based gap junctions during cervical cancer pathogenesis. The data obtained in the present study could also have major implications for understanding how intercellular communication via gap junctions is lost in other cancer types in which ITCH is overexpressed and displays oncogenic features [82].

## Materials and methods

### Cell culture

HeLa cells negative for Cx43 and HeLa-Cx43 cells were kindly gifted by Professor Klaus Willecke (University of Bonn, Germany) and have been characterized previously by Elfgang et al. [83]. C33A cells were obtained from American Type Culture Collection (ATCC; Manassas, VA, USA). Cells were cultured in Dulbecco’s modified Eagle’s medium (Invitrogen, San Diego, CA, USA) supplemented with 10% (v/v) fetal bovine serum (Gibco BRL Life Technologies, Inchinnan, UK) and 1% L-glutamine (Sigma-Aldrich, St. Louis, MO, USA) at 37 °C in a humidified incubator containing 5% CO_2_.

### Reagents and antibodies

Bafilomycin A1 (B1793), chloroquine (C6628), protease inhibitor cocktail (P8340), phosphatase inhibitor cocktails II (P5726) and III (P0044), and Lucifer Yellow (L0259) were from Sigma-Aldrich. Protein A Sepharose CL-4B was purchased from GE Healthcare Life Sciences (Chicago, IL, USA). Tetramethylrhodamine-conjugated dextran (D1817) and ProLong Gold Antifade Mountant (P36930) were from Thermo Fisher Scientific. Rabbit IgG (2729) was obtained from Cell Signaling Technology (Danvers, MA, USA). Anti-Cx43 antibody was from Sigma-Aldrich (C6219; immunofluorescence 1:500 dilution; western blotting 1:5000 dilution; immunoprecipitation 1:10 dilution) [84]. Mouse anti-β-actin (A2228; 1:5000 dilution) antibody was from Sigma-Aldrich. Mouse anti-ubiquitin antibody P4D1 (3936; 1:1500 dilution) was purchased from Cell Signaling Technology. Mouse anti-ITCH antibody (611,198; 1:1000 dilution) was obtained from BD Transduction Laboratories (Franklin Lakes, NJ, USA). Rabbit anti-ITCH antibody (HPA021126; 1:1000 dilution) was obtained from Sigma-Aldrich. Mouse anti-HA antibody (6E2; western blotting 1:1000 dilution; immunofluorescence 1:100 dilution) was from Cell Signaling Technology. Mouse anti-LAMP1 (H4A3; 1:100 dilution) antibody was from Developmental Studies Hybridoma Bank (Iowa City, IA, USA). Alexa555-conjugated wheat germ agglutinin (WGA) (W32464), Alexa405-conjugated goat anti-mouse IgG antibody (A31553; 1:1000 dilution), Alexa555-conjugated goat anti-mouse IgG antibody (A21424, 1:1000 dilution), Alexa488-conjugated goat anti-rabbit IgG antibody (A11034; 1:1000 dilution), Alexa594-conjugated donkey anti-mouse IgG antibody (A21203, 1:1000 dilution) and Alexa647-conjugated donkey anti-mouse IgG antibody (A31571, 1:000 dilution) were from Thermo Fisher Scientific (Waltham, MA, USA). Horseradish peroxidase (HRP)-conjugated donkey anti-mouse IgG antibody (715-035-150; 1:2000 dilution) was purchased from Jackson Immunoresearch Laboratories, Inc. (West Grove, PA, USA). HRP-conjugated goat anti-rabbit IgG antibody (170–6515; 1:2000 dilution) was obtained from Bio-Rad (Hercules, CA, USA). IRDye 680RD donkey anti-mouse (926-68072; 1:15000) and IRDye 800CW donkey anti-rabbit (926-32213; 1:15000) antibodies were from LI-COR Biotechnology (Lincoln, NE, USA). The RC DC protein assay was from Bio-Rad (5,000,121).

### Plasmids

The expression plasmid pcDNA I/Neo (Invitrogen) encoding human Cx43 was kindly provided by Klaus Willecke (University of Bonn, Germany) [83]. The expression plasmid pcDNA3.4-TOPO encoding HA-ITCH-WT was prepared by Thermo Fisher by using the GeneArt technology platform. The P283A and C830A point mutations in Cx43 and HA-ITCH cDNA, respectively, were generated by using QuikChange II XL site-directed mutagenesis kit (Agilent, Santa Clara, CA, USA). The constructs were confirmed by DNA sequencing.

### DNA and siRNA transfection

Cells growing in 35-mm Petri dishes were transfected with plasmids or siRNA by using Lipofectamine 2000 (Thermo Fisher Scientific) following the manufacturer’s instructions, as described previously [85]. For DNA transfection, the cells were transfected 5 h after seeding and assayed 48 h after transfection. For siRNA transfection, the cells were transfected 5 h after seeding, retransfected 48 h after the first transfection, and assayed 96 h after the first transfection. The final siRNA concentration used was 80 nM. The six siRNA oligonucleotides targeted against *ITCH* were obtained from Qiagen or Thermo Fisher Scientific and had the following sequences: siRNA #1: 5´-CGGGCGAGUUUACUAUGUATT-3´ (Qiagen; Hs_ITCH_1); siRNA #2: 5´- AGAGCUAUGAGCAACUGAATT-3´ (Qiagen; Hs_ITCH_3); siRNA #3: 5´- AACTACCCGTTCATTATATAA-3´ (Qiagen; Hs_ITCH_9); siRNA #4: 5´-CCACAATAGAAGAACTACCACCCTAT-3´ (Thermo Fisher Scientific; ITCHHSS130351); siRNA #5: 5´- GGACCACAGAAATTCTGCATTGAAA-3´ (Thermo Fisher Scientific; ITCHHSS130353); siRNA #6: 5´- CATACAAGAGCTATGAGCAACTGAA-3´ (Thermo Fisher Scientific; ITCHHSS188825). Stealth RNAi Negative Control (Medium GC; Thermo Fisher Scientific) was used as the siRNA oligonucleotide control construct.

### Confocal fluorescence microscopy

Cells were washed twice with Dulbecco′s phosphate-buffered saline (DPBS) containing Ca^2+^ and Mg^2+^, fixed with 3.8% paraformaldehyde in DPBS for 15 min at room temperature, and washed twice with DPBS. In experiments involving labeling of cells with WGA, the cells were incubated with 5 µg/ml Alexa555-conjugated WGA diluted in Hank’s balanced salt solution for 10 min at 37 °C prior to fixation. For staining against Cx43 alone or in combination with HA, the cells were permeabilized by incubating them with 0.1% Triton X-100 in PBS for 30 min. The cells were washed twice with DPBS and blocked for 1 h in 5% (w/v) non-fat dry milk in PBS containing 0.1% Tween. The cells were incubated with the appropriate primary antibodies diluted in 5% (w/v) non-fat dry milk in PBS containing 0.1% Tween either for 1 h or overnight, depending on the antibody used. The cells were washed three times with PBS containing 0.1% Tween and incubated for 1 h with secondary antibodies conjugated to Alexa488, Alexa555, or Alexa594 in appropriate combinations diluted in 5% (w/v) non-fat dry milk in PBS containing 0.1% Tween. The cells were washed three times with PBS containing 0.1% Tween, and nuclei were stained by using 10 µg/ml Hoechst 33342 diluted in PBS. The cells were washed once in PBS and then mounted on object slides by using ProLong Gold Antifade Mountant (ThermoFisher Scientific). For co-staining against LAMP1 and Cx43, the cells were permeabilized by incubating them with 0.05% (w/v) saponin in PBS for 10 min at 4 °C and then incubated overnight with the appropriate primary antibodies diluted in PBS containing 0.05% (w/v) saponin. The cells were washed three times with PBS and incubated for 1 h with secondary antibodies conjugated to Alexa405 and Alexa488 diluted in PBS containing 0.05% (w/v) saponin. The cells were washed three times in PBS and then mounted on object slides by using ProLong Gold Antifade Mountant (Thermo Fisher Scientific). Images were acquired with an LSM 710 META confocal microscope (Carl Zeiss Inc., Oberkochen, Germany) with a Plan Apochromat 63 × 1.4 NA oil immersion objective (Carl Zeiss Inc.). The ZEN software (2009 edition) was used for acquisition, and images were assembled and processed by using Adobe Photoshop CS4. Quantitative image analysis was done by using NIS-elements AR software (Nikon Instruments Inc., Melville, NY). Briefly, nuclei, cells, and Cx43-containing structures were segmented. Segmented cells were filtered for expression of Cx43 and/or HA-ITCH, in accordance with the experimental setup, and sometimes also manually filtered for the presence of plasma membrane contact between appropriately transfected adjacent cells before further analyses. Cx43 structures were scored as intracellular or gap junctions located in the plasma membrane, based on WGA staining, and the gap junction area was measured and summed per cell. For each experiment, images from three biological replicates, each including at least 10 images per condition, were analyzed and the data was subjected to statistical analyses.

### SDS-PAGE and western blotting

Cells were washed once with PBS and collected in 300 µl of SDS sample buffer (10 mm Tris (pH 6.8), 15% w/v glycerol, 3% w/v SDS, 0.01% w/v bromophenol blue, and 5% v/v 2-mercaptoethanol). The samples were sonicated and heated at 95 °C for 5 min and subjected to 7.5% or 10% SDS-PAGE. Protein concentrations were measured using the RC DC protein assay from Bio-Rad. For chemiluminescence detection, the samples were transferred to nitrocellulose membranes and for fluorescence detection they were transferred to low-fluorescence polyvinylidene difluoride membranes. For chemiluminescence detection, the membranes were developed by using SuperSignal West Dura Extended Duration Substrate (Thermo Fisher Scientific) or Lumiglo (EMD Millipore, Billerica, MA, USA) and imaged by using the ChemiDoc XRS+ (Bio-Rad) or ChemiDoc MP systems (Bio-Rad). For fluorescence detection, the ChemiDoc MP or Odyssey imaging systems (Li-COR) were used. Image processing (brightness and contrast adjustments) and quantification of protein band intensity was performed using ImageJ 1.48v or Image Lab v6.0.1 software.

### Immunoprecipitation

Immunoprecipitation of Cx43 was performed as described previously [85]. Briefly, cells growing in 35-mm or 100-mm Petri dishes were washed once with ice-cold PBS and collected in lysis buffer (PBS, 10% glycerol, 0.25% sodium deoxycholate, 0.45% sodium lauroyl sarcosine, 20 mm*N*-ethylmaleimide, 2 mm EDTA) containing protease inhibitor cocktail (Sigma; 100 µl/ml) and phosphatase inhibitor cocktails II and III (Sigma; both 10 µl/ml). For precleaning, the lysates were incubated with protein A-Sepharose beads (Invitrogen) at 4 °C under constant rotation for 30 min. Beads were pelleted by centrifugation and the resulting supernatant was collected. To covalently link anti-Cx43 antibodies to protein A-Sepharose beads, we incubated protein A-Sepharose beads in immunoprecipitation coupling buffer (dH_2_O, 100 mm NaHCO_3_, 50 mm NaCl, and 1 mM phenylmethanesulfonyl fluoride (PMSF; pH 8.3)) for 30 min, and then incubated them together with anti-Cx43 antibodies for 2 h under gentle rotation. The protein A-Sepharose beads bound to anti-Cx43 antibodies were washed twice with 10 volumes of 0.2 m borate buffer (pH 9) and then added the cross-linking agent, dimethyl pimelimidate dihydrochloride to a final concentration of 20 mm. The cross-linking reaction was ended after 30 min by washing the protein A-Sepharose beads linked to anti-Cx43 antibodies three times in 10 volumes of 0.2 M ethanolamine (pH 8) followed by incubation in 10 volumes of 0.2 M ethanolamine for 2 h with continuous gentle rotation. The ethanolamine was removed, and the protein A-Sepharose beads linked to anti-Cx43 antibodies were resuspended in ice-cold PBS containing 1 mM PMSF. Equal amounts of protein A-Sepharose beads linked to anti-Cx43 were added to each precleared cell lysate sample, followed by incubation for 2 h at 4 °C under constant rotation. Cell lysates incubated with protein A-Sepharose beads linked to rabbit IgG were used as negative controls. The samples were centrifuged at 2200*g* for 5 min at 4 °C, and the pellet was washed five times with ice-cold lysis buffer added with 20 mM *N*-ethylmaleimide. Following the last wash, the pellet was resuspended in 40 µl western sample buffer and then heated at 95 °C for 5 min. The immunoprecipitation samples were subjected to SDS-PAGE and western blotting.

### Quantitative scrape loading-dye transfer assay

Gap junction intercellular communication was measured by performing the quantitative scrape loading-dye transfer assay, as described previously [86]. Briefly, confluent HeLa-Cx43 cells growing in 35-mm Petri dishes were washed twice with PBS. Two milliliters of PBS (without Ca^2+^ and Mg^2+^) containing 0.05% (w/v) Lucifer Yellow (Sigma) and 0.05% (w/v) tetramethylrhodamine-conjugated Dextran (Thermo Fisher) was added to the dishes. A surgical scalpel was used to make four to five scratches through the cell monolayer in each dish, and the cells were left at room temperature for 3.5 min. The Lucifer Yellow solution was removed and the cells were rinsed four times with PBS. The cells were fixed in 3.8% paraformaldehyde in PBS and mounted with a glass coverslip by using ProLong Gold Antifade Mountant (Thermo Fisher Scientific). Images were acquired by using an LSM 710 META confocal microscope (Carl Zeiss Inc., Oberkochen, Germany) with a Plan Apochromat 10×/0.45 NA objective (Carl Zeiss Inc). The ZEN software (2009 edition) was used for acquisition and images were assembled and processed with Adobe Photoshop CS4. The relative level of gap junctional intercellular communication in HeLa-Cx43 cells transfected with control siRNA and in HeLa-Cx43 cells transfected with siRNA targeting ITCH was determined, by using ImageJ software, as the relative area of dye-coupled cells. To this end, two ImageJ macros were made, for Lucifer Yellow and tetramethylrhodamine-conjugated Dextran images, respectively. The images were converted to black and white images and binarized. Subsequently, the net area of cells having received Lucifer Yellow via gap junctions was calculated in each image by subtracting the area of the cells positive for tetramethylrhodamine-conjugated Dextran from the area of cells positive for Lucifer Yellow.

### Statistical analysis

Data were statistically analyzed by using SigmaPlot 14.5. Statistically significant differences between groups were determined by one-way analysis of variance with post hoc analysis by using the Student-Newman-Keuls multiple comparison test.

### Electronic supplementary material

Below is the link to the electronic supplementary material.

Supplementary Material 1

## Data Availability

The datasets generated during and/or analyzed during the current study are available from the corresponding author on reasonable request.
