# Supplementary material for: The E3 ubiquitin ligase ITCH negatively regulates intercellular communication via gap junctions by targeting connexin43 for lysosomal degradation
Source: Cell Mol Life Sci. 2024 Apr 10;81(1):171. doi: 10.1007/s00018-024-05165-8 (PMC11006747; doi:10.1007/s00018-024-05165-8)
Supplement: Supplementary file 1 — Supplementary Material 1 [file 18_2024_5165_MOESM1_ESM.pdf]

SUPPLEMENTARY FIGURE LEGENDS

Supplementary Figure S1. Effect of knockdown of ITCH on the Cx43 protein level. (A) HeLa-Cx43 cells were transfected with six different siRNA sequences against ITCH (#1-6), with a final concentration of 80 nM, for 96 hours. Cell lysates were prepared and equal amounts of total protein were subjected to SDS-PAGE. Cx43, ITCH, NEDD4, SMURF2, and β-actin were detected by western blotting. β-actin was used as a loading control. Molecular mass in kDa is indicated. **(B)** The intensities of the Cx43 bands on western blots obtained in A were quantified and normalized to the β-actin level. The values are shown as percentages of the control and are the means ± SD of three independent experiments. **(C)** The intensities of ITCH, NEDD4 and SMURF2 signals based on the western blot data obtained in A were quantified and normalized to the β-actin level. The values are shown as percentages of the control and are the means ± SD of three independent experiments. *$p<0.05$, one-way ANOVA.

Supplementary Figure S2. Effect of knockdown of ITCH on the size and number of Cx43-based gap junctions. (A) HeLa-Cx43 cells were transfected with control siRNA or with six different siRNA sequences against ITCH (#1-6), with a final concentration of 80 nM, for 96 hours. The siRNA sequence ITCH#1 corresponds to the siRNA sequence used in the experiments whose results are presented in Figure 1. The cells were fixed and stained with anti-Cx43 (green) antibodies followed by Alexa488-conjugated secondary antibodies. The plasma membrane was stained with Alexa555-conjugated WGA (red). Nuclei were stained with Hoechst 33342 (blue). The cells were visualized by confocal fluorescence microscopy. Scale bar, 10 µm, applies to all images. **(B,C)** Quantification of the area of Cx43-based gap junctions per cell (B) and number of Cx43-based gap junctions per cell (C) based on confocal fluorescence microscopy images in A. Values shown are the means ± S.E.M. of three independent experiments.

Supplementary Figure S3. Effect of knockdown of ITCH on Cx43 ubiquitination. (A) HeLa-Cx43 cells were transfected with control siRNA or with an siRNA sequence against ITCH (corresponding to siRNA #2 in Suppl. Fig. 1), as indicated, for 96 hours. Cell lysates were then subjected to immunoprecipitation by using anti-Cx43 antibodies and equal amounts of immunoprecipitates were subjected to SDS-PAGE. Ubiquitinated Cx43 was detected with

western blotting by using anti-ubiquitin antibodies (upper panel). The blot was stripped and reprobed with anti-Cx43 antibodies (lower panel). Also shown is the relative expression of Cx43, ITCH, and β-actin in cell lysates prior to immunoprecipitation (input). Molecular mass in kDa is indicated. **(B)** Quantification of ubiquitinated Cx43 based on the data obtained in A. For each lane, the level of ubiquitin immunoreactivity was normalized to the level of Cx43 immunoreactivity in the immunoprecipitates. Values shown are the means ± S.E.M. of eight independent experiments. *p<0.05.

Supplementary Figure S4. Effect of bafilomycin A1 and chloroquine on the ITCH-induced loss of Cx43 protein. (A) C33A cells were transfected with HA-ITCH-WT for 48 hours. The cells were treated with chloroquine (100 µM) for the last 18 hours of the transfection. The cells were fixed and stained with anti-Cx43 (green) and anti-HA (red) antibodies, followed by Alexa488- and Alexa555-conjugated secondary antibodies. Nuclei were stained with Hoechst 33342 (blue). The cells were visualized by confocal fluorescence microscopy. Scale bar, 10 µm. **(B)** HeLa-Cx43 cells were transfected with HA-ITCH-WT for 48 hours. The cells were treated with DMSO (vector) or bafilomycin A1 (BafA1; 200 nM), as indicated, for the last 18 hours of the transfection. The cells were fixed and stained with anti-Cx43 (green) and anti-HA (grey) antibodies followed by Alexa488- and Alexa647-conjugated secondary antibodies. The plasma membrane was stained with Alexa555-conjugated WGA (red). Nuclei were stained with Hoechst 33342 (blue). The cells were visualized by confocal fluorescence microscopy. Scale bar, 10 µm, applies for all images. **(C)** HeLa cells negative for Cx43 were transfected with Cx43 alone or in combination with HA-ITCH-WT for 48 hours, as indicated. The cells were treated with DMSO (vector), bafilomycin A1 (BafA1; 200 nM) or chloroquine (100 µM) for the last 18 hours of the transfection, as indicated. The cells were fixed and stained with anti-Cx43 (green) and anti-HA (red) antibodies, followed by Alexa488- and Alexa555-conjugated secondary antibodies. Nuclei were stained with Hoechst 33342 (blue). The cells were visualized by confocal fluorescence microscopy. **(D)** *Lower panel:* HeLa cells negative for Cx43 were transfected with empty vector for 48 hours. For the last 18 hours of the transfection, the cells were treated with DMSO (vector) or bafilomycin A1 (BafA1; 200 nM), as indicated. Cell lysates were then prepared and equal amounts of total cell protein were subjected to SDS-PAGE. ITCH and β-actin were detected by western blotting. Molecular mass in kDa is indicated. *Upper panel:* The intensities of the ITCH bands on western blots

were quantified and normalized to the level of β-actin. Values shown are the means ± S.D. of three independent experiments. n.s., not significant. **(E)** *Lower panel:* HeLa cells negative for Cx43 were transfected with empty vector for 48 hours. For the last 18 hours of the transfection, the cells were treated with DMSO (vector) or chloroquine (Chl; 100 µM), as indicated. Cell lysates were then prepared and equal amounts of total cell protein were subjected to SDS-PAGE. ITCH and β-actin were detected by western blotting. Molecular mass in kDa is indicated. *Upper panel:* The intensities of the ITCH bands on western blots were quantified and normalized to the level of β-actin. Values shown are the means ± S.D. of three independent experiments. n.s., not significant.

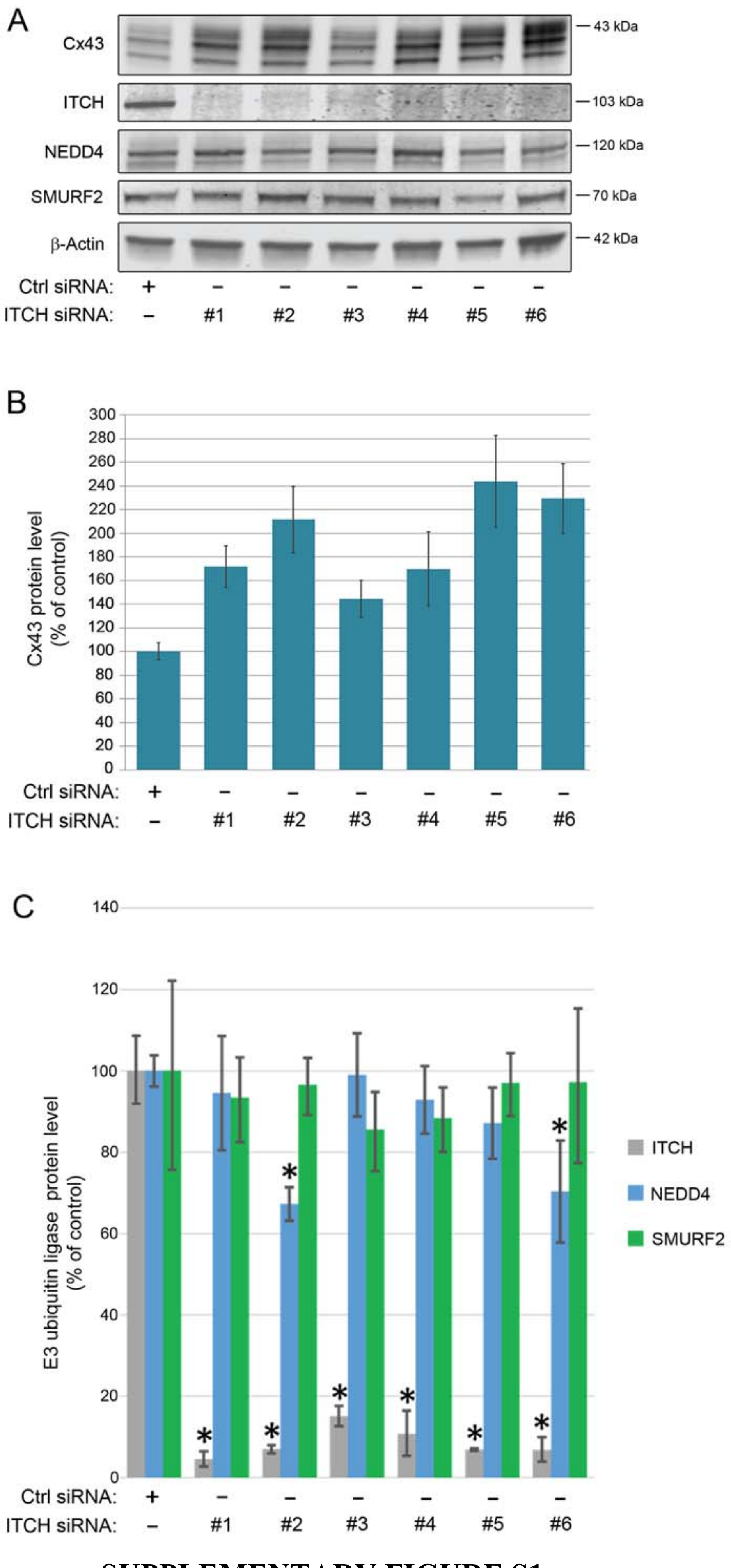

SUPPLEMENTARY FIGURE S1

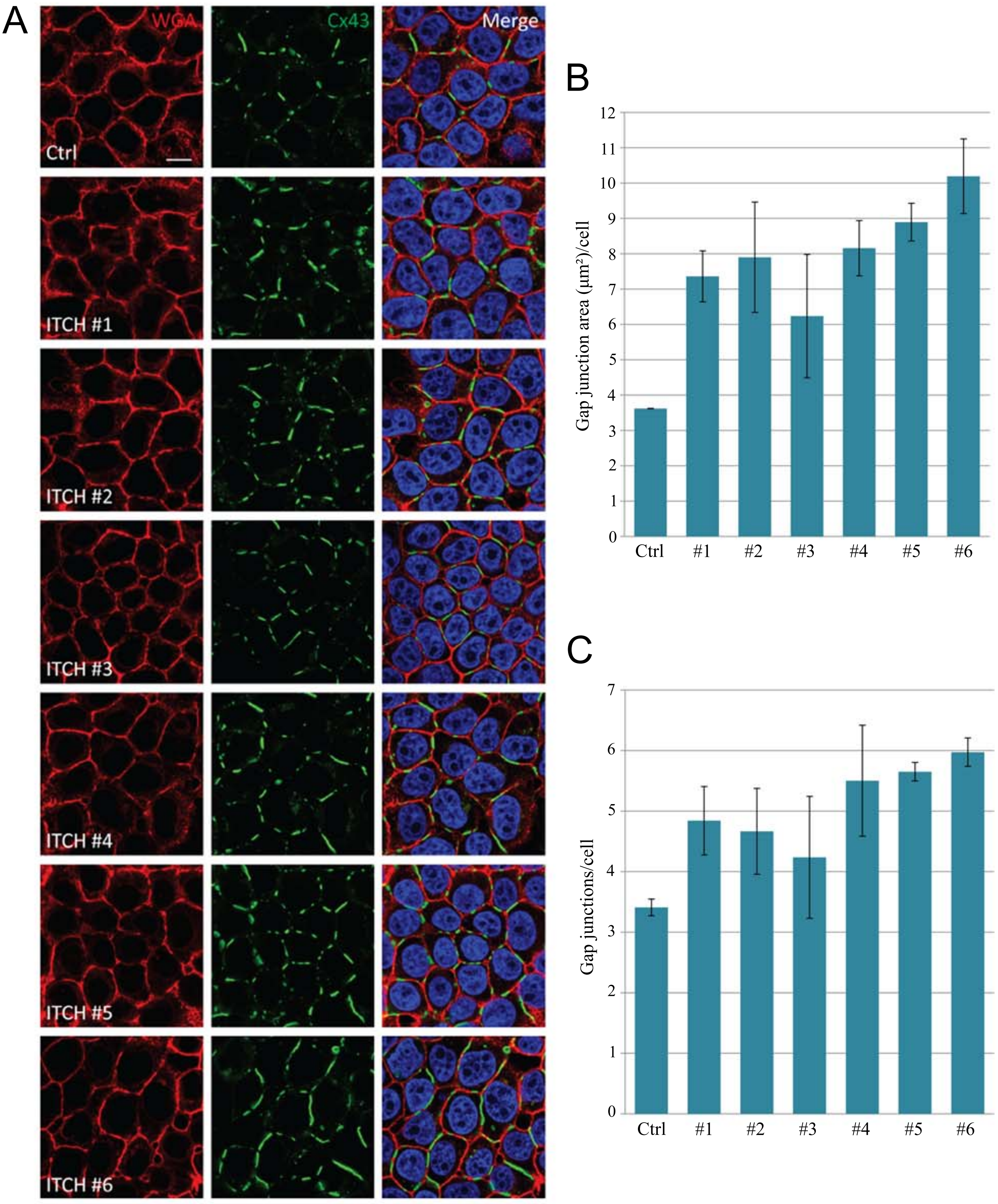

SUPPLEMENTARY FIGURE S2

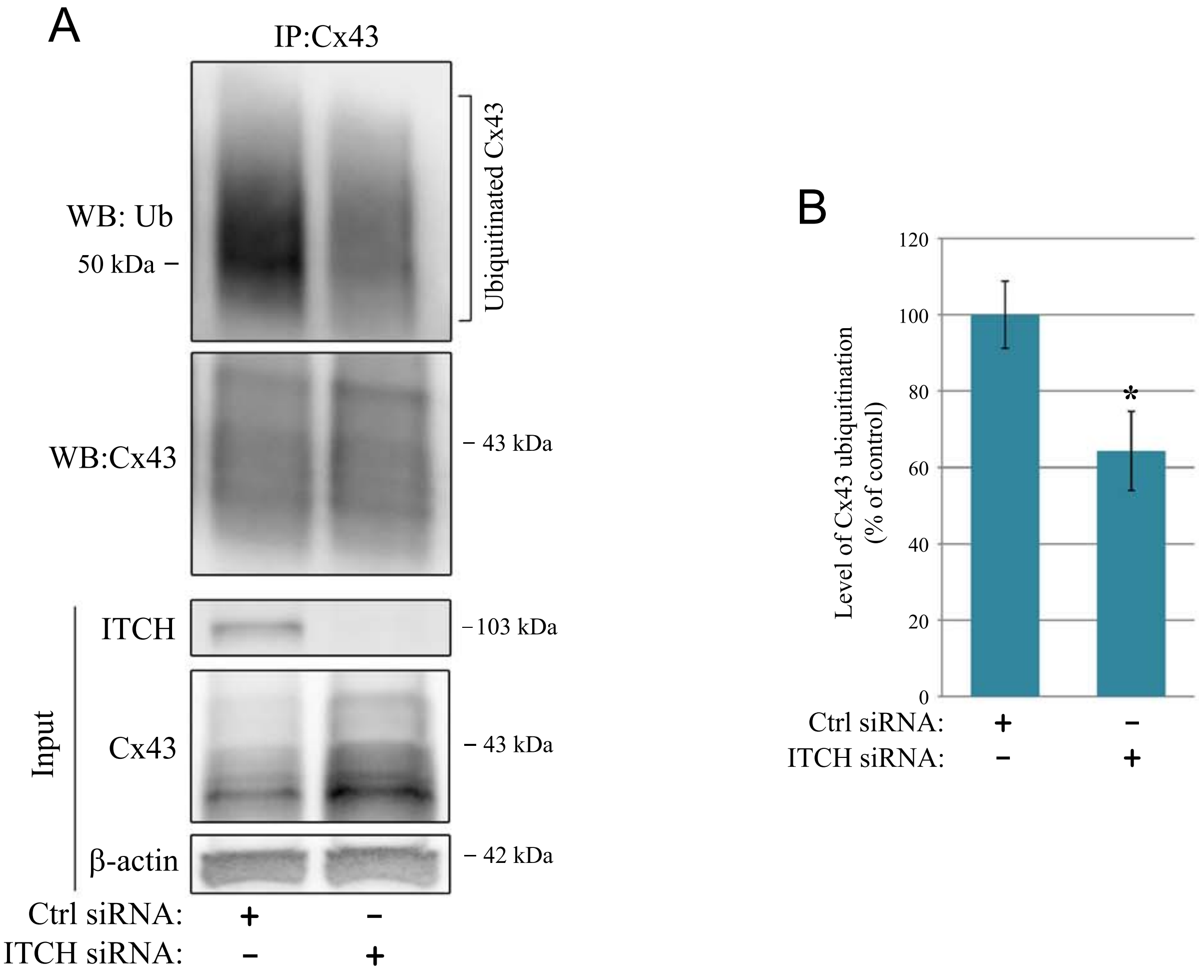

SUPPLEMENTARY FIGURE S3

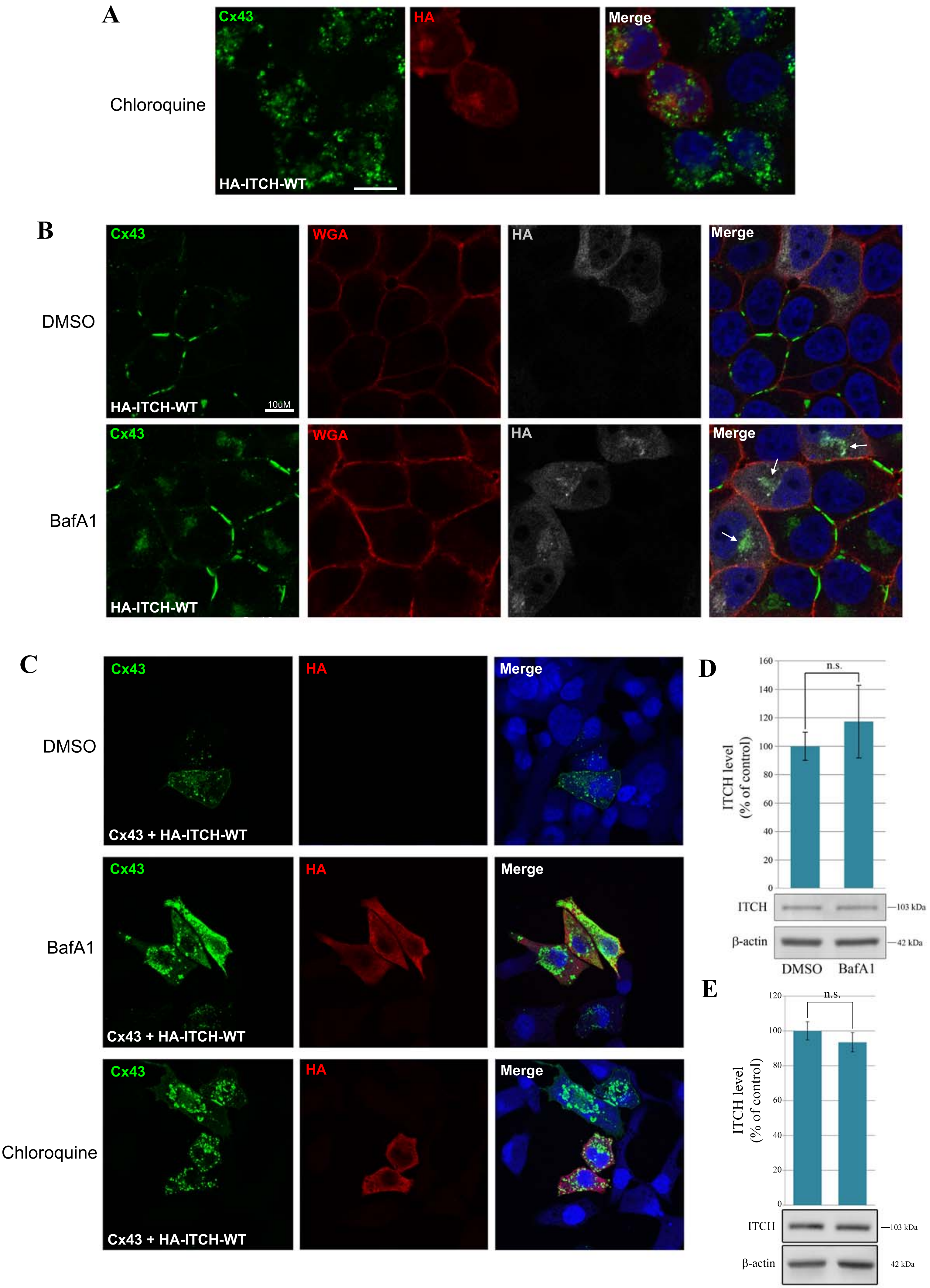

SUPPLEMENTARY FIGURE S4
